# Supplementary material for: Mouse Heterochromatin Adopts Digital Compaction States without Showing Hallmarks of HP1-Driven Liquid-Liquid Phase Separation
Source: Mol Cell. 2020 Apr 16;78(2):236–249.e7. doi: 10.1016/j.molcel.2020.02.005 (PMC7163299; doi:10.1016/j.molcel.2020.02.005)
Supplement: Document S1. Figures S1–S7 and Tables S1–S5 [file mmc1.pdf]

Molecular Cell, Volume *78*

Supplemental Information

Mouse Heterochromatin Adopts Digital Compaction States without Showing Hallmarks of HP1-Driven Liquid-Liquid Phase Separation

Fabian Erdel, Anne Rademacher, Rifka Vlijm, Jana Tünnermann, Lukas Frank, Robin Weinmann, Elisabeth Schweigert, Klaus Yserentant, Johan Hummert, Caroline Bauer, Sabrina Schumacher, Ahmad Al Alwash, Christophe Normand, Dirk-Peter Herten, Johann Engelhardt, and Karsten Rippe

Supplemental Figures

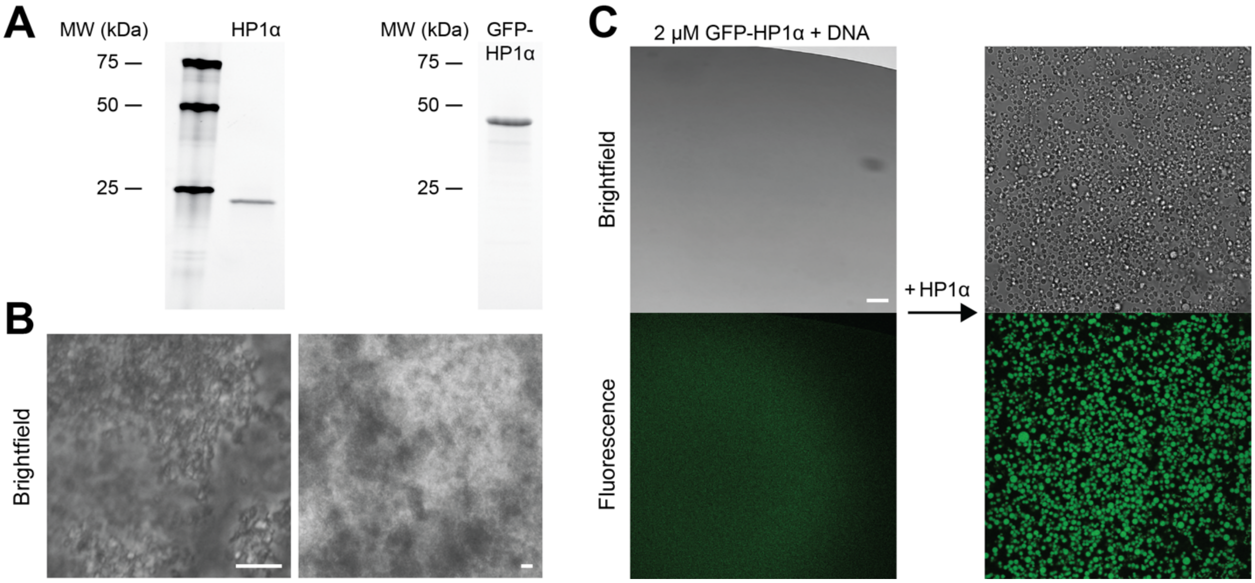

Figure S1. Characterization of recombinant mouse HP1α. Related to Fig. 1.

(**A**) SDS polyacrylamide gel electrophoresis of purified mouse HP1α and GFP-HP1α.
(**B**) Irregular structures in mixtures of HP1α and DNA that formed in addition to droplets, especially at very high HP1α concentrations. Scale bars, 10 μm.
(**C**) Left: No droplets formed in a mixture of 2 μM GFP-HP1α and saturating amounts of DNA. Right: Upon addition of a 180 μM solution of untagged HP1α to the same mixture under the microscope, green fluorescent droplets formed. The final concentrations after HP1α addition were 45 μM untagged HP1α and 1.5 μM GFP-HP1α. All components were dissolved in storage buffer (STAR Methods). Scale bar, 10 μm.

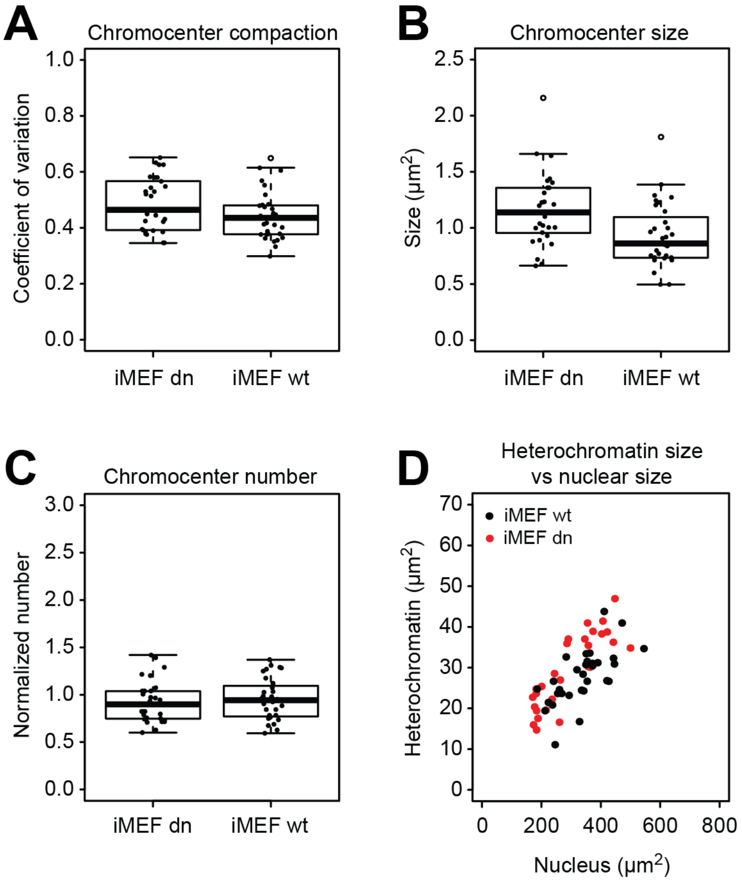

Figure S2. Compaction, size and number of chromocenters. Related to Fig. 2.

(**A**) Compaction of chromocenters assessed by the coefficient of variation of DAPI signals. A similar approach has previously been used to measure heterochromatin compaction (Casas-Delucchi et al., 2012).
(**B**) Size of chromocenters determined from DAPI signals.
(**C**) Number of chromocenters per 10 μm^2 nuclear area. As the number of chromocenters was positively correlated with the size of cell nuclei, the normalization was used to make the results independent of nuclear size.
(**D**) The area occupied by heterochromatin scales with the size of the nucleus. This is in contrast to the inverse scaling that has been reported for nucleoli in early *C. elegans* embryos (Weber and Brangwynne, 2015), which is expected for organelles formed by LLPS (assuming that the number of molecules that drive LLPS is the same in each nucleus).

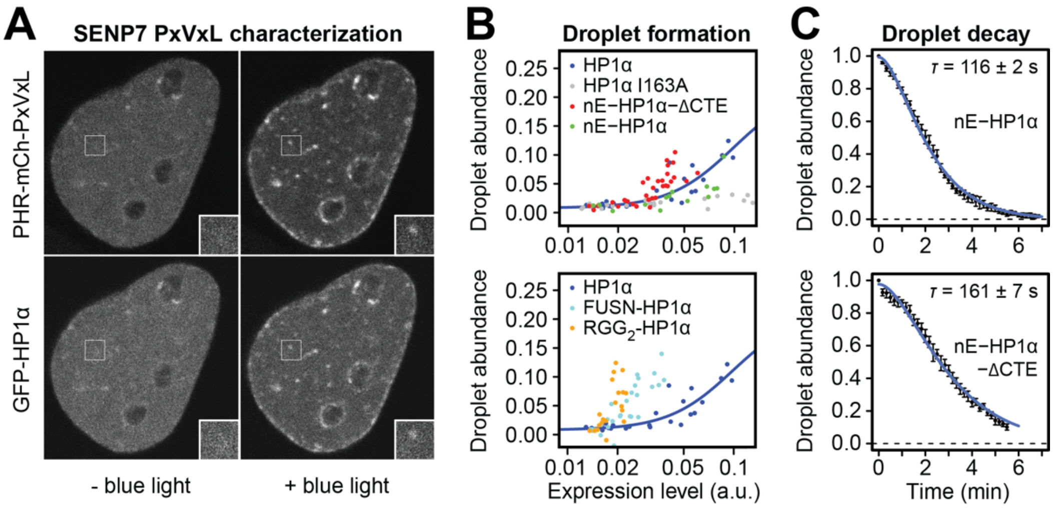

Figure S3. Properties of optodroplets containing different proteins. Related to Fig. 3.

(**A**) Confocal microscopy images of a representative U2OS cell that shows colocalization of GFP-HP1α and the PHR-mCherry-tagged PxVxL module of SENP7. Both proteins were located at perinucleolar regions before illumination with blue light (left) and assembled into optodroplets after illumination (right, see inset for a magnified optodroplet). Based on the GFP-HP1α signal in both images, heterochromatin regions already present before blue light exposure can be distinguished from light-induced optodroplets.
(**B**) Capacity of droplet formation for fusions of PHR-mCherry with the indicated proteins.
(**C**) Dissociation kinetics of optodroplets containing fusions of PHR-mCherry with the phosphomimetic HP1 variants nE-HP1α (top) and nE-HP1α-ΔCTE (bottom). Error bars represent s.e.m. from at least 10 replicates. Errors for half-lives represent standard fit errors.

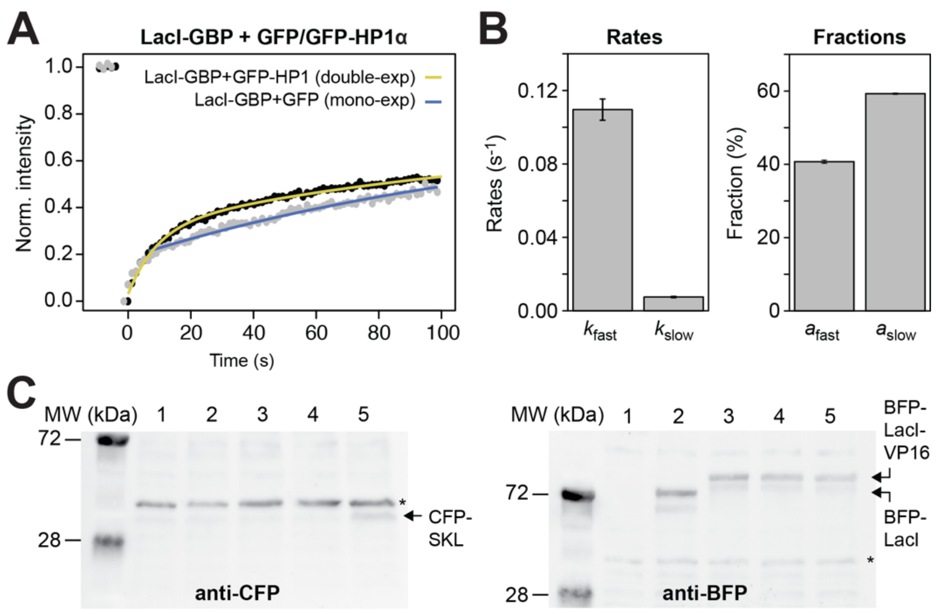

Figure S4. Protein exchange and activity at the reporter array. Related to Fig. 4.

(**A**) GFP bound to *lac*O arrays via LacI-GBP (GFP binding protein) was bleached to measure the exchange kinetics of directly recruited GFP-proteins. The data for GFP (gray points) and GFP-HP1α (black points) were globally fitted with a mono-exponential function for GFP (blue curve) and a double-exponential function for GFP-HP1α (yellow curve), with the rate for GFP being kept identical to the slow rate for GFP-HP1α. The early time points for GFP were not considered for the fit because they contain a contribution from diffusion of LacI-GBP•GFP, while the contribution from slow exchange of LacI-GBP•GFP at the *lac*O array is most prominent for later time points.

(**B**) Parameters obtained from the global fit in the previous panel. Error bars represent standard fit errors. k_{fast} is the rate for the fast component that is specific to GFP-HP1α; k_{slow} is the rate for the slower component that is present in both recovery curves; a_{fast} and a_{slow} are the sizes of the fast and the slow fraction for GFP-HP1α.

(**C**) Western Blot analysis of U2OS reporter cells under different conditions. The CFP-SKL protein visible in the left blot (probed with an antibody against GFP/CFP) is encoded by the reporter adjacent to the *lac*O/*tet*O sites (see (Janicki et al., 2004) for a detailed description of the construct). The right blot was probed with an antibody against BFP and shows the different transiently expressed BFP-fusions. The lanes correspond to the following conditions: 1: untransfected cells; 2: BFP-LacI-transfected cells; 3-5: BFP-LacI-VP16-transfected cells, with doxycycline and with light (3), with doxycycline and without light (4) and without doxycycline and without light (5). Asterisks indicate nonspecific bands.

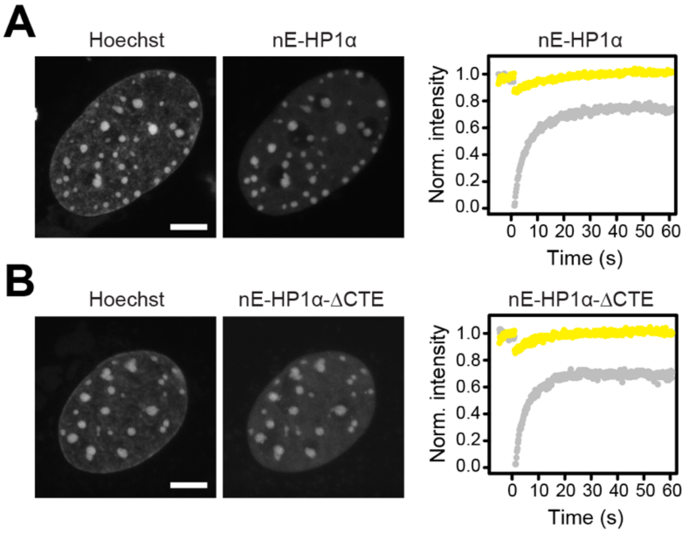

Figure S5. Internal mixing of phosphomimetic HP1α variants in chromocenters. Related to Fig. 5.

Localization and half-chromocenter bleach for the phosphomimetic HP1 variants GFP-nE-HP1α (panel **A**; *n* = 11 cells for the bleach experiment) and GFP-nE-HP1α-ΔCTE (panel **B**; *n* = 16 cells for the bleach experiment). No anti-correlation between the bleached and the non-bleached half was observed. Accordingly, the phosphomimetic HP1α variants behaved similarly to GFP-HP1α and displayed no preferential internal mixing in chromocenters. Bleach experiments were conducted in cells without Hoechst. Scale bars, 5 µm.

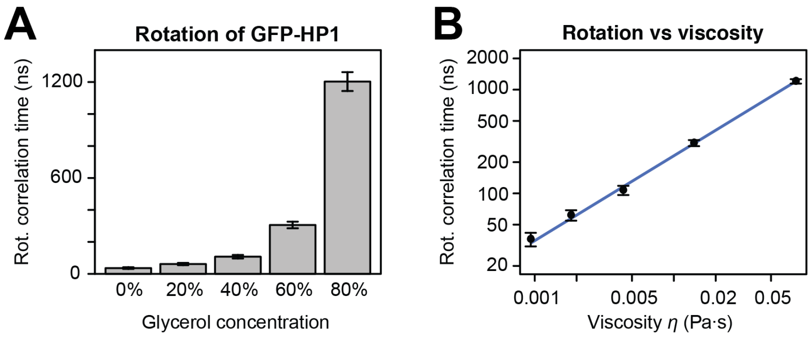

Figure S6. Relationship between viscosity and GFP-HP1 rotational correlation times *in vitro*. Related to Fig. 6.

Pol-FCS experiments were conducted with purified GFP-HP1 at a concentration of 50 nM in glycerol-water mixtures with the indicated glycerol concentrations.
(**A**) Rotational correlation times for GFP-HP1 in glycerol-water mixtures. Error bars represent standard fit errors.
(**B**) Relationship between GFP-HP1 rotational correlation times from the previous panel and the viscosity of the respective glycerol-water mixture taken from the literature (Cheng, 2008). The blue line is a fit with the equation $\tau_R/\tau_{R,0} = (\eta/\eta_0)^q$ (Lavalette et al., 1999), yielding $q = 0.82 \pm 0.01$ (the error is the standard fit error).

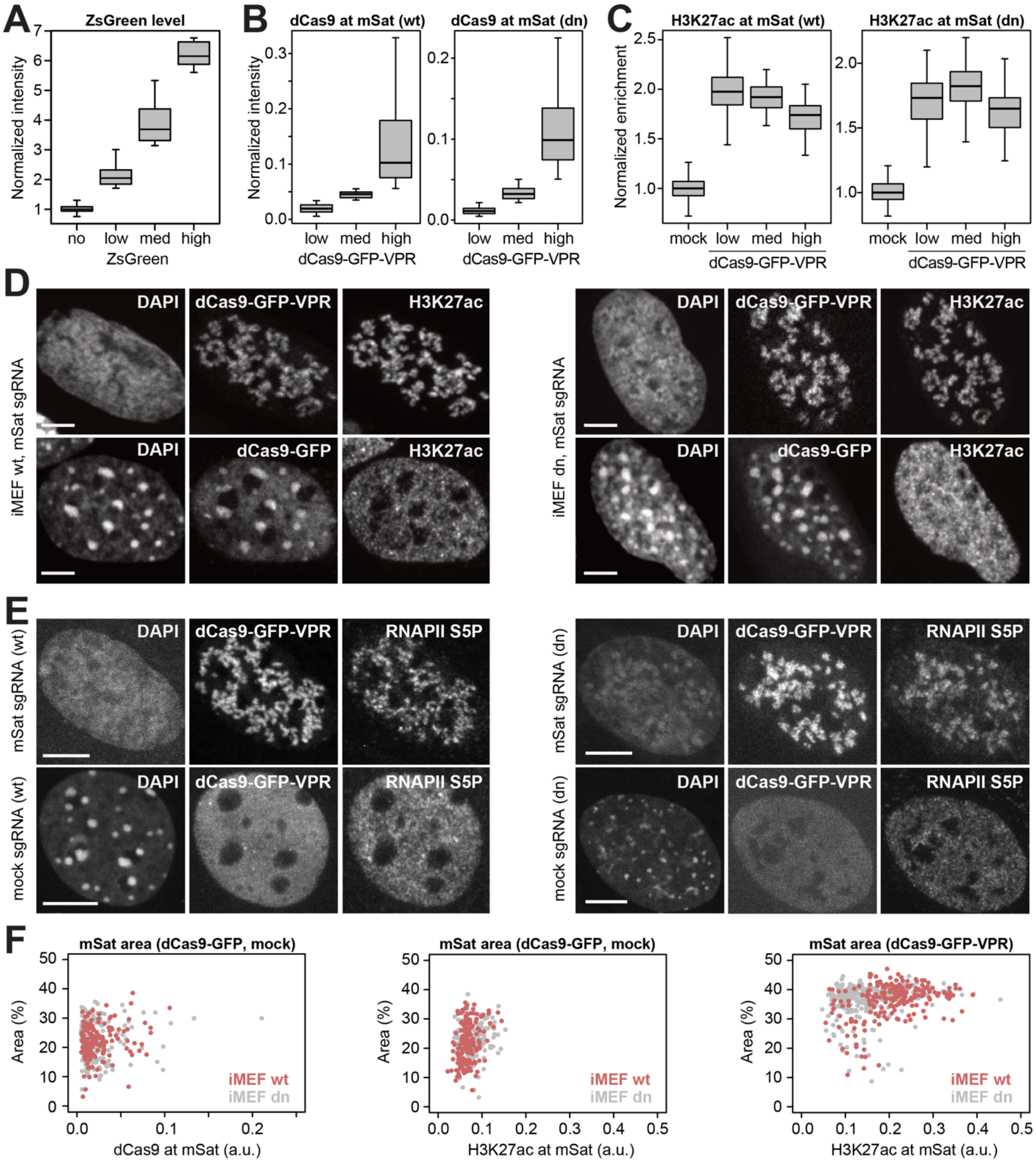

Figure S7. Properties of iMEF cells exposed to different perturbations. Related to Fig. 7.

(**A**) Expression level of ZsGreen for the individual groups of cells analyzed in **Fig. 7C**.
(**B**) Amount of dCas9 bound at major satellites for the individual groups of cells analyzed in **Fig. 7F** and **7I**.
(**C**) Amount of H3K27ac at major satellites for the individual groups of cells analyzed in **Fig. 7F** and **7I**.
(**D**) DAPI, dCas9 and H3K27ac signals for the cells shown in **Fig. 7E** (left) and for iMEF dn cells expressing the same constructs for comparison (right). Scale bars, 5 µm.
(**E**) DAPI, dCas9-GFP-VPR and RNA polymerase II phosphorylated at serine 5 (RNAPII S5P) signals for the cell shown in **Fig. 7H** (top left), for an iMEF wt cell expressing a mock sgRNA instead of an mSat sgRNA (bottom left), and for iMEF dn cells expressing the same constructs for comparison (right). Scale bars, 5 µm.
(**F**) Relationship between chromocenter size and dCas9-GFP (left) or H3K27ac (center/right) at major satellites in iMEF wt (red) and iMEF dn (gray) cells expressing dCas9-GFP (left/center) or dCas9-GFP-VPR (right).

Supplemental Tables

Fit parameter	HP1α	GFP-HP1α
c_{sat} (μM)	45 ± 4	23 ± 2
n	1.7 ± 0.1	1.5 ± 0.1
a	1.5 ± 0.1	

Table S1. Fit results for turbidity curves. Related to Fig. 1.

Parameters for Hill equation fits (STAR Methods) to the turbidity curves for HP1α and GFP-HP1α in **Fig. 1B**. Errors represent standard fit errors. The upper plateau (*a*) was globally fitted for both proteins, increasing the robustness of the fit.

Fit parameter	HP1α, iMEF wt	H3K9me3, iMEF wt	HP1α, iMEF dn	H3K9me3, iMEF dn
a_1	0.60 ± 0.05	0.74 ± 0.01	1	1
a_2	0.40 ± 0.05	0.26 ± 0.01	-	-
λ_1 (nm)	51 ± 9	60 ± 1	73 ± 2	75 ± 1
λ_2 (nm)	318 ± 8	329 ± 7	-	-
n_1	0.76 ± 0.05	1.15 ± 0.02	0.68 ± 0.01	0.79 ± 0.01
n_2	2.35 ± 0.16	2.15 ± 0.07	-	-

Table S2. Fit results for image correlation spectroscopy functions. Related to Fig. 2.

Parameters for exponential fits (STAR Methods) to the normalized image correlation spectroscopy functions for HP1α and H3K9me3 shown in **Fig. 2C**. Errors represent standard fit errors. The correlation lengths λ_i reflect the radii of the structures in the images and were multiplied by a factor of 2 to obtain the diameters reported in **Fig. 2D**. The parameter values for λ and n depend on each other, which means that the uncertainty for both parameters is larger than reflected by the reported error. The parameters for the second component (a_2, λ_2, n_2) depend on the segmentation settings and should be interpreted with caution.

Fit parameter	GFP-HP1α	MECP2-GFP	GFP-NCL	GFP-NPM	H2B-GFP
τ_D (s)	64 ± 1	233 ± 7	9.8 ± 0.6	18.9 ± 1.4	> 1,000
p	≥ 100	≥ 100	0.64 ± 0.04	≤ 0.01	-

Table S3. Fit results for half-chromocenter and half-nucleolus FRAP. Related to Fig. 5.

Parameters for the confined diffusion fits (STAR Methods) to the FRAP curves shown in **Fig. 5D-H**. Note that τ_D is differently defined than the diffusion time in many other unconfined FRAP models. Permeability values p below 0.01 and above 100 could not be determined as curves showed only subtle changes in these parameter regimes. The permeability for H2B-GFP could not be determined because the protein did not show sufficient diffusive motion during the course of the experiment. Errors represent standard fit errors.

Fit parameter	Cytoplasm	Nucleoplasm	Chromocenter
τ_R (ns)	74 ± 7	117 ± 9	111 ± 8
τ_T (μs) *	0.80 ± 0.09	0.80 ± 0.09	0.80 ± 0.09
τ_{D1} (ms) *	0.33 ± 0.03	0.33 ± 0.03	0.33 ± 0.03
τ_{D2} (ms)	-	35 ± 11	89 ± 18
f_R	0.50 ± 0.03	0.67 ± 0.03	0.80 ± 0.04
f_T *	0.25 ± 0.02	0.25 ± 0.02	0.25 ± 0.02
f_1	1	0.78 ± 0.03	0.67 ± 0.03
α_1	0.70 ± 0.04	0.6	0.6
α_2	-	1.14 ± 0.29	1.23 ± 0.24

Table S4. Fit results for Pol-FCS in living cells. Related to Fig. 6.

Parameters for the rotational-translational diffusion fits (STAR Methods) to the Pol-FCS curves for GFP-HP1α shown in **Fig. 6D** and **6E**. Errors represent standard fit errors.
* f_T, τ_T and τ_{D1} were fitted globally.

Fit parameter	0%	20%	40%	60%	80%
τ_R (ns)	36 ± 5	61 ± 7	107 ± 11	304 ± 20	1199 ± 59
τ_T (μs) *	0.79 ± 0.14	0.79 ± 0.14	0.79 ± 0.14	0.79 ± 0.14	0.79 ± 0.14
τ_D (ms)	0.66 ± 0.09	1.66 ± 0.19	1.25 ± 0.16	2.66 ± 0.37	2.00 ± 0.27
f_R	0.68 ± 0.06	0.53 ± 0.04	0.77 ± 0.04	0.85 ± 0.03	1.14 ± 0.03
f_T *	0.13 ± 0.01	0.13 ± 0.01	0.13 ± 0.01	0.13 ± 0.01	0.13 ± 0.01
α	0.63 ± 0.04	0.64 ± 0.04	0.64 ± 0.04	0.54 ± 0.03	0.54 ± 0.03

Table S5. Fit results for Pol-FCS in glycerol-water mixtures. Related to Fig. 6 and Fig. S6.

Parameters for the rotational-translational diffusion fits (STAR Methods) to the Pol-FCS curves for GFP-HP1 in glycerol-water mixtures shown in **Fig. 6G**. Errors represent standard fit errors.
* f_T and τ_T were fitted globally.
