# Supplementary material for: Mouse Heterochromatin Adopts Digital Compaction States without Showing Hallmarks of HP1-Driven Liquid-Liquid Phase Separation
Source: Mol Cell. 2020 Apr 16;78(2):236–249.e7. doi: 10.1016/j.molcel.2020.02.005 (PMC7163299; doi:10.1016/j.molcel.2020.02.005)
Supplement: Document S2. Article plus Supplemental Information [file mmc3.pdf]

Molecular Cell

Article

Mouse Heterochromatin Adopts Digital Compaction States without Showing Hallmarks of HP1-Driven Liquid-Liquid Phase Separation

Graphical Abstract

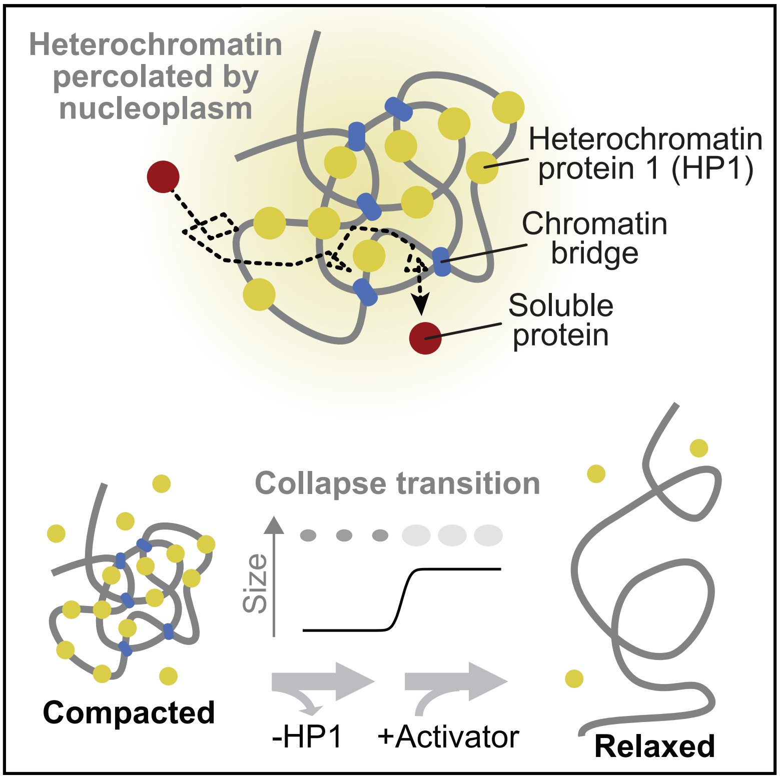

Highlights

- HP1 has only a weak capacity to form droplets in living cells
- Size, accessibility, and compaction of heterochromatin foci are independent of HP1
- Heterochromatin compaction is "digital" and can toggle between two distinct states
- Methodological framework to assess hallmarks of phase separation in living cells

Authors

Fabian Erdel, Anne Rademacher, Rifka Vlijm, ..., Dirk-Peter Herten, Johann Engelhardt, Karsten Rippe

Correspondence

fabian.erdel@ibcg.biotoul.fr (F.E.),
karsten.rippe@dkfz.de (K.R.)

In Brief

Mouse cells package heterochromatin into compact foci. Erdel et al. show that these foci lack hallmarks of liquid droplets and rather resemble collapsed polymer globules. Their size, accessibility, and compaction are independent of HP1. They can adopt two distinct folding states that possibly represent the fundamental modes of chromatin compaction.

Erdel et al., 2020, Molecular Cell *78*, 236–249
April 16, 2020 © 2020 The Author(s). Published by Elsevier Inc.
https://doi.org/10.1016/j.molcel.2020.02.005

CellPress

Molecular Cell
Article

Mouse Heterochromatin Adopts Digital Compaction States without Showing Hallmarks of HP1-Driven Liquid-Liquid Phase Separation

Fabian Erdel,[1,2,8,*] Anne Rademacher,[2] Rifka Vlijm,[3,7] Jana Tünnermann,[2] Lukas Frank,[2] Robin Weinmann,[2] Elisabeth Schweigert,[2] Klaus Yserentant,[4] Johan Hummert,[4] Caroline Bauer,[2] Sabrina Schumacher,[2] Ahmad Al Alwash,[2] Christophe Normand,[1] Dirk-Peter Herten,[4,5,6] Johann Engelhardt,[3] and Karsten Rippe[2,*]

[1]LBME, Centre de Biologie Intégrative (CBI), CNRS, UPS, Toulouse, France
[2]Division of Chromatin Networks, German Cancer Research Center (DKFZ) and Bioquant, Heidelberg, Germany
[3]Department of Optical Nanoscopy, Max Planck Institute for Medical Research, Heidelberg, Germany
[4]Department for Physical Chemistry, Heidelberg University, Heidelberg, Germany
[5]Institute of Cardiovascular Sciences, College of Medical and Dental Sciences and School of Chemistry, University of Birmingham, Birmingham, UK
[6]Centre of Membrane Proteins and Receptors (COMPARE), Universities of Birmingham and Nottingham, United Kingdom
[7]Present address: Faculty of Science and Engineering, University of Groningen, Groningen, the Netherlands
[8]Lead Contact
*Correspondence: fabian.erdel@ibcg.biotoul.fr (F.E.), karsten.rippe@dkfz.de (K.R.)
https://doi.org/10.1016/j.molcel.2020.02.005

SUMMARY

The formation of silenced and condensed heterochromatin foci involves enrichment of heterochromatin protein 1 (HP1). HP1 can bridge chromatin segments and form liquid droplets, but the biophysical principles underlying heterochromatin compartmentalization in the cell nucleus are elusive. Here, we assess mechanistically relevant features of pericentric heterochromatin compaction in mouse fibroblasts. We find that (1) HP1 has only a weak capacity to form liquid droplets in living cells; (2) the size, global accessibility, and compaction of heterochromatin foci are independent of HP1; (3) heterochromatin foci lack a separated liquid HP1 pool; and (4) heterochromatin compaction can toggle between two "digital" states depending on the presence of a strong transcriptional activator. These findings indicate that heterochromatin foci resemble collapsed polymer globules that are percolated with the same nucleoplasmic liquid as the surrounding euchromatin, which has implications for our understanding of chromatin compartmentalization and its functional consequences.

INTRODUCTION

Cells partition their genome into distinct chromatin domains with specific functions. Some of them form micrometer-sized chromatin subcompartments in three-dimensional nuclear space (Cavalli and Misteli, 2013; Kundaje et al., 2015; van Steensel and Furlong, 2019). A prominent example is that of the dense heterochromatin foci at silenced pericentric satellite repeats, which are also called chromocenters because of their intense DAPI staining (Probst and Almouzni, 2008). Chromocenters contain elevated levels of DNA methylation, repressive histone modifications like trimethylation of histone H3 at lysine 9 (H3K9me3), and a specific set of proteins, including HP1, that can bind to H3K9me3 via its chromodomain (Bannister et al., 2001). The repressive heterochromatin state can spread to genomic sequences in proximity to pericentric repeats, leading to a phenomenon called position effect variegation (Elgin and Reuter, 2013). Because the accurate position and size of heterochromatin domains is critical for proper cell function (Fodor et al., 2010), it is crucial to understand how chromatin partitioning is faithfully accomplished.

Heterochromatin formation involves recruitment of HP1, which can form bridges between nucleosomes (Hiragami-Hamada et al., 2016; Kilic et al., 2018; Machida et al., 2018) and can undergo liquid-liquid phase separation (LLPS) (Larson et al., 2017; Strom et al., 2017). Both of these properties can, in principle, induce formation of compact heterochromatin domains, as reviewed recently (Erdel and Rippe, 2018). In brief, chromatin bridging can potentially promote formation of ordered and collapsed chromatin globules. These domains would be percolated by the nucleoplasmic liquid but be separated from the surrounding chromatin in the sense that loci within the globule contact each other more frequently than they contact loci outside of it (Cook and Marenduzzo, 2018; Jost et al., 2017; Leibler, 1980; MacPherson et al., 2018; Michieletto et al., 2016; Nicodemi and Pombo, 2014; Nuebler et al., 2018). LLPS of HP1 can potentially form a liquid droplet that encloses heterochromatic sequences. It would separate heterochromatin from the surrounding chromatin by a "boundary" that selectively regulates access of molecules at the interface based on their chemical properties (Banani et al., 2017; Larson et al., 2017; Strom et al., 2017; Taylor et al., 2019). Both mechanisms are not mutually exclusive because interactions among HP1 molecules

 © 2020 The Author(s). Published by Elsevier Inc.
This is an open access article under the CC BY license (http://creativecommons.org/licenses/by/4.0/).

might drive both chromatin bridging and LLPS. Moreover, weak interactions among lowly abundant HP1 molecules might lead to weak bridging among heterochromatin loci without generating collapsed globules or liquid droplets.

Whether heterochromatin is established by droplet formation of HP1, collapse into a chromatin globule, or weak bridging without globule or droplet formation has a number of functional implications. The droplet model predicts that the size of chromocenters increases when the total cellular HP1 level increases, whereas the HP1 concentration inside chromocenters remains constant, a behavior known as ‘‘concentration buffering’’ (Banani et al., 2017). Accordingly, heterochromatin spreading might result from increasing HP1 levels, whereas heterochromatin maintenance might rely on the buffered HP1 concentration in chromocenters. Conversely, the chromatin globule model predicts that the size of chromocenters is not directly coupled to total cellular HP1 levels, whereas the HP1 concentration inside chromocenters follows the total cellular HP1 level. We refer to this behavior as ‘‘size buffering.’’ In this scenario, heterochromatin spreading and maintenance would have to be regulated by other means. For decreasing cellular HP1 levels, droplets should dissolve when the critical concentration is reached (Banani et al., 2017), whereas collapsed globules should transition into a distinct decondensed state (Leibler, 1980; Michieletto et al., 2016). Thus, the globule model predicts switch-like behavior with ‘‘digital’’ compaction states (compacted or decompacted, rarely intermediate). In contrast, the droplet model is compatible with digital or ‘‘analog’’ states depending on the coupling between compaction state and droplet size. Another key hallmark of liquid droplets is preferential internal mixing; because of the dynamic attractive protein-protein interactions that drive LLPS, phase-separating proteins should preferentially move within the droplet. This internal protein pool might have specific properties (e.g., particular posttranslational modifications), creating a chemical environment that is distinct from its surroundings. Attractive protein-protein interactions should also tend to increase the apparent viscosity inside the droplet (Hyman et al., 2014). In particular, interactions that depend on the relative orientation of neighboring phase-separating proteins should decrease their rotational diffusion coefficient. In this manner, the kinetics of binding and enzymatic reactions would be locally modulated in the droplet. In contrast, proteins in a chromatin globule would not experience retardation by increased viscosity or retention by a boundary with interfacial resistance, although diffusion barriers created by obstacles might obstruct molecular transport.

It is currently unclear how HP1 drives heterochromatin compartmentalization in living cells and which of the functional consequences above arise from it. To address this question, here we assessed key biophysical properties of chromocenters and the associated heterochromatin proteins in mouse fibroblasts. We compared the capacity of HP1 to form droplets *in vitro*, in the nucleoplasm and when tethered to chromatin, and found that it does not form stable droplets in living cells. By studying molecular transport in chromocenters and by following their response to forced activation, we found that chromocenters resemble collapsed chromatin globules. Their global compaction, accessibility, and size was independent of HP1. Depending on the presence of transcriptional activators, they toggled between two digital chromatin compaction states. These two states might represent the fundamental compaction modes of chromatin that control long-range chromatin contacts and accessibility to nucleoplasmic factors.

RESULTS

Mouse HP1α and GFP-HP1α Form Droplets in the Presence of DNA *In Vitro*

It has recently been reported that *Drosophila* HP1a and human HP1α can form liquid droplets *in vitro*, which for human HP1α is promoted by phosphorylation, addition of DNA, or removal of salt (Larson et al., 2017; Strom et al., 2017; Wang et al., 2019; Zhang et al., 2019). To test the ability of mouse HP1 to form droplets *in vitro*, we expressed and purified recombinant mouse HP1α and GFP-HP1α (Figure S1A) and mixed both proteins with a concentrated solution of fragmented salmon sperm DNA. At high protein concentrations, both HP1α and GFP-HP1α formed droplets (Figure 1A) as well as more irregular structures, which might correspond to assemblies of coagulated droplets (Figure S1B). To quantitate the propensity of both proteins to associate into droplets and possibly other structures that are large enough to scatter light, we measured the turbidity of DNA/HP1 mixtures in dependence of the HP1 concentration, similar to a previously used approach (Larson et al., 2017). The turbidity of both DNA/HP1α and DNA/GFP-HP1α mixtures increased with protein concentration, with half-saturation concentrations of 45 μM for HP1α and 23 μM for GFP-HP1α (Figure 1B; Table S1). Next we prepared mixtures of both proteins at different stoichiometries and tested whether droplets formed in these mixtures. We observed green fluorescent droplets but no colorless droplets (Figure 1C), indicating that GFP-HP1α and HP1α do not form separate droplet populations but rather co-localize in the same ones. Increasing fractions of GFP-HP1α seemed to favor droplet formation over formation of coagulated structures. To test more directly whether GFP-HP1α enters HP1α droplets, we prepared a mixture of DNA and 2 μM GFP-HP1α, which is well below the half-saturation concentration for droplet formation, and added untagged HP1α to it to reach a final HP1α concentration of 45 μM (Figure S1C). Upon HP1α addition, we observed green fluorescent droplets, indicating that GFP-HP1α enters HP1α droplets without dissolving them. We conclude that HP1α and GFP-HP1α have a similar propensity to form large structures when mixed with DNA and that GFP-HP1α can be used to label droplets formed by untagged HP1α.

Chromocenters Contain Clusters with Moderate HP1 Enrichment

The half-saturation concentrations of more than 40 μM determined for mammalian HP1α droplet formation above and in a previous study (Larson et al., 2017) are considerably higher than the average HP1α concentration of ~1 μM that we had measured in mouse fibroblasts (Müller-Ott et al., 2014). Accordingly, we wondered whether chromocenters contain small substructures with locally elevated HP1 concentrations and visualized HP1α and H3K9me3 after immunostaining in immortalized

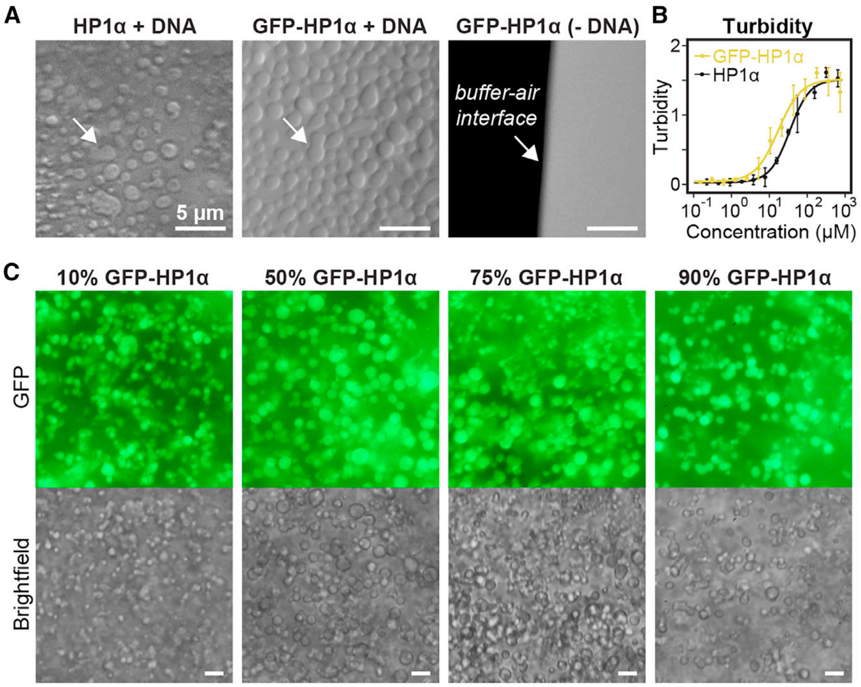

Figure 1. Droplet Formation of Recombinant Mouse HP1α in the Presence of DNA

(A) Visualization of droplet formation by HP1α and GFP-HP1α when mixed with DNA. Arrows in the left and center panel highlight *bona fide* fusion intermediates. Scale bars, 5 μm. See also Figure S1.

(B) Turbidity measurements for HP1α and GFP-HP1α in the presence of saturating amounts of DNA. Error bars represent SD from 3 replicates. The lines are Hill functions fitted to the data, assuming the same plateau value for both proteins. Fit parameters are listed in Table S1.

(C) Visualization of droplet formation in mixtures of HP1α and GFP-HP1α (in the presence of DNA). The concentrations of GFP-HP1α amounted to 16 μM, 80 μM, 120 μM, and 144 μM (left to right). The total HP1 concentration in the samples was kept at ~180 μM. Scale bars, 5 μm.

mouse embryonic fibroblasts (iMEFs) by stimulated emission depletion (STED) nanoscopy. Chromocenters in wild-type (WT) iMEF cells showed robust enrichment of DAPI, HP1α, and H3K9me3 signals (Figures 2A and 2B). In contrast, iMEF cells with double knockout of the *Suv39h1* and *Suv39h2* genes that encode H3K9 methyltransferases (*Suv39h* dn) lacked H3K9me3 and HP1α enrichment at chromocenters, as shown previously (Peters et al., 2001). Nevertheless, *Suv39h* dn cells retained distinct chromocenters, as reflected by the DAPI signal. The number, size, and compaction of chromocenters in *Suv39h* dn cells were similar to those in WT cells (Figure S2), indicating that their formation did not critically depend on HP1α or H3K9me3 enrichment. Next we assessed the internal structure of chromocenters. Neither the HP1α nor the H3K9me3 signal were homogenously distributed within chromocenters but rather formed a clustered pattern (see magnified panels in Figures 2A and 2B). To quantify the properties of these clusters, we segmented chromocenters in the DAPI channel and analyzed the HP1 and H3K9me3 distributions by image correlation spectroscopy (Figure 2C). The inverse amplitude of the resulting correlation functions is a measure of the abundance of clusters, and their width is a measure of the characteristic cluster size (Petersen et al., 1993). Figure 2D shows the abundance and size of clusters for the different conditions obtained by fitting a generic function to the correlation curves (STAR Methods; Table S2). HP1 and H3K9me3 clusters had a characteristic size of ~100–150 nm in both WT and *Suv39h* dn cells (~7–10 pixels in the STED images), whereas WT cells showed an additional component reflecting larger structures (Table S2). The correlation amplitudes yielded a 2- to 3-fold enrichment of clusters in WT cells compared with *Suv39h* dn cells, which is similar to the enrichment of average intensities we measured previously in mouse fibroblasts (Müller-Ott et al., 2014). In addition, we found that the intensities of most pixels in chromocenters of WT cells were contained within a relatively narrow band around the median, with the maximum intensity of some pixels being 2–3 times larger than the median (Figure 2D). We conclude that chromocenters are not completely homogeneous but contain clusters enriched for HP1α and/or H3K9me3, with local HP1α concentrations reaching up to ~3 μM when equating the median intensity with the previously measured ~1 μM concentration (Müller-Ott et al., 2014). These estimates suggest that the HP1α concentration in heterochromatin is well below the half-saturation concentration for *in vitro* droplet formation reported above.

HP1 Promotes Droplet Formation but Does Not Form Stable Droplets in the Nucleoplasm

Next we tested whether HP1 droplets are stable in the nucleoplasm of living cells. To nucleate droplets, we used the recently developed optodroplet system (Shin et al., 2017). Optodroplets employ the N-terminal photolyase homology region (PHR) of cryptochrome 2, which switches into a "sticky" conformation upon illumination with blue light (Figure 3A). In this conformation, PHR has the tendency to form droplets, which is enhanced when PHR is fused to a protein that drives droplet formation. Because the conformational switch is reversible, it can be tested whether nucleated droplets are stable without the contribution from light-induced PHR interactions. The resulting optodroplets are nucleated throughout the entire nucleoplasm and not only at heterochromatin. Accordingly, the droplet formation capacity of HP1 can be assessed independently of heterochromatin-specific processes like HP1 binding to pericentromeres, which might confound the result. We fused HP1α to PHR-mCherry and expressed the fusion protein in iMEF cells. PHR-mCherry-HP1α localized to chromocenters in the absence of light and formed droplets when illuminated with blue light (Figure 3B). Next we transfected PHR-mCherry-HP1α into immortalized human osteosarcoma (U2OS) cells, which lack pronounced heterochromatin foci and thus provide a homogeneous background

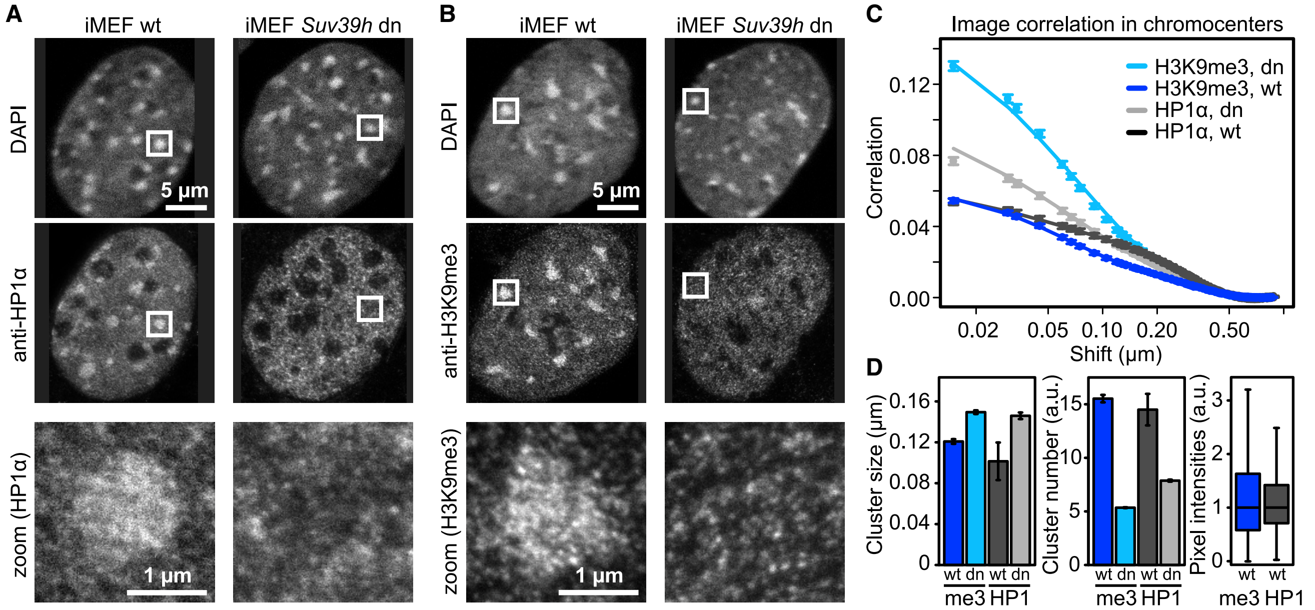

Figure 2. Internal Structure of Chromocenters

(A) Distribution of HP1α in WT and *Suv39h* dn iMEF cells, visualized by immunostaining and STED nanoscopy. DNA was stained with DAPI and imaged by conventional confocal microscopy. The images in the first two rows have the same magnification.

(B) Same as (A) but for H3K9me3.

(C) Image correlation spectroscopy analysis of HP1α and H3K9me3 signals in chromocenters of iMEF WT and *Suv39h* dn cells. A total of 18 (H3K9me3, WT), 19 (H3K9me3, dn), 24 (HP1α, WT), and 14 (HP1α, dn) cells were analyzed. Error bars represent SEM. Solid lines represent fit functions (STAR Methods).

(D) Quantitation of cluster size (left) and abundance (center) from fitting the correlation functions shown in (C) and pixel intensity distribution in chromocenters (right). Correlation functions for WT cells contained an additional large component and were fitted with double-exponential functions (STAR Methods; Table S2). Error bars represent standard fit errors. See also Figure S2 for a quantitative analysis of the compaction, number, and size of chromocenters in WT and *Suv39h* dn iMEF cells.

(Figure 3C). Similar to iMEF cells, U2OS cells expressing PHR-mCherry-HP1α displayed clearly visible droplets upon illumination with blue light that were absent from cells expressing PHR-mCherry at similar levels (Figure 3D). To quantify the droplet formation capacity of HP1α, we determined the relative saturation concentration from the relationship between droplet abundance and expression level (Figure 3E; Figure S3B; STAR Methods). The following proteins were used for comparison: (1) PHR-mCherry; (2) the monomeric variant HP1α I163A, which is not expected to promote droplet formation (Larson et al., 2017); (3) phosphomimetic variants of HP1α (Larson et al., 2017); (4) the PxVxL module of SENP7, which interacts with HP1α (Romeo et al., 2015) and can be used to study HP1-containing optodroplets without overexpressing any HP1 fusion (Figure S3A); (5) nucleolin (NCL), an abundant nucleolar protein with disordered domains that is involved in organization and, potentially, phase separation of the nucleolus (Caudron-Herger et al., 2015; Emmott and Hiscox, 2009); (6) a fusion of HP1α with the N-terminal intrinsically disordered region of FUS (FUSN) that promotes droplet formation (Bracha et al., 2018; Shin et al., 2017); and (7) a fusion of HP1α with the arginine/glycine-rich RGG domains of LAF-1 that also promote droplet formation (Schuster et al., 2018). WT HP1α promoted droplet formation compared with HP1α I163A and PHR-mCherry (Figure 3E). The phosphomimetic variants of HP1α behaved similarly as WT HP1α (Figure S3B). However, SENP7 PxVxL, NCL, as well as fusions of HP1α with FUSN and RGG_2 displayed a much stronger capacity to promote droplet formation (Figure 3E; Figure S3B). Next we evaluated the stability of optodroplets after switching off the blue light. If optodroplets are mainly stabilized by PHR-PHR interactions, then their lifetime should correspond to ~1–2 min in the absence of blue light (Shin et al., 2017), and interactions among candidate proteins fused to PHR should increase this value. Accordingly, we measured the lifetimes of optodroplets containing fusions of PHR-mCherry with HP1α, HP1β, HP1γ, MECP2 (another heterochromatin marker protein), SENP7 PxVxL, NCL, nucleophosmin (NPM, a nucleolar protein linked to LLPS; Feric et al., 2016), FUSN-HP1α, RGG_2-HP1α, and a nuclear localization sequence (NLS) as a control (Figure 3F). For HP1γ and NLS, lifetimes of 76–89 s were obtained, whereas optodroplets for HP1α, HP1β, SENP7 PxVxL, MECP2, NCL, and NPM were slightly more stable and persisted for 106–148 s. FUSN-HP1α and RGG_2-HP1α optodroplets exhibited the longest lifetimes, reaching more than 16 min for some of them (Video S1). Again, the phosphomimetic variants of HP1α behaved similarly as WT HP1α (Figure S3C). These results suggest that HP1α, HP1β, SENP7 PxVxL, MECP2, NCL, NPM, and/or their interactions partners might exhibit multivalent interactions that can promote the formation and transient stabilization of liquid droplets, albeit on very short timescales.

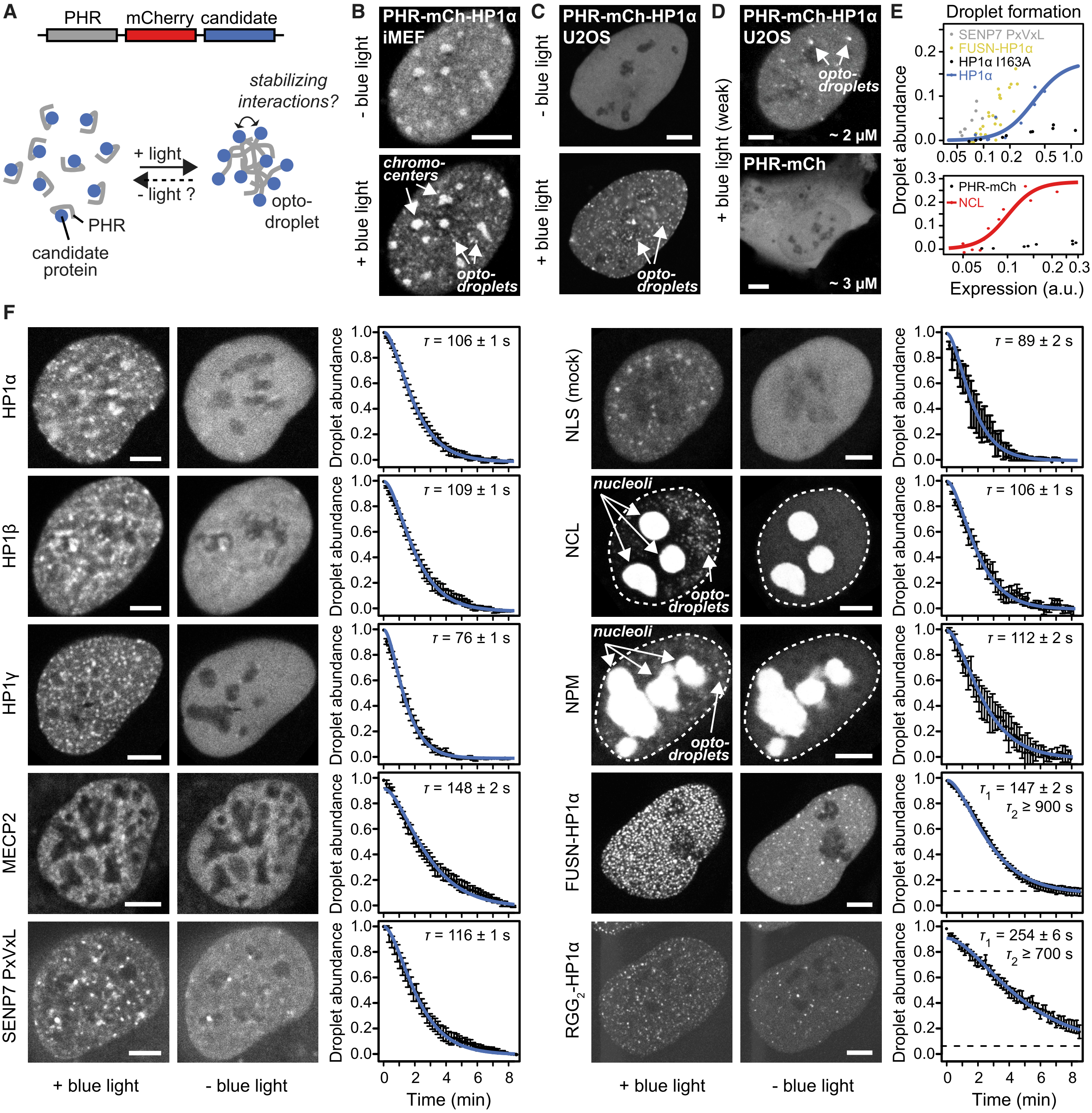

Figure 3. Formation and Stability of Optodroplets in Living Cells

(A) Schematic representation of the optodroplet system and the experimental design. A protein of interest ("candidate") is fused to PHR-mCherry, and its ability to form and stabilize droplets is evaluated by switching blue light on/off.

(B) Localization and droplet formation of PHR-mCherry-HP1α in iMEF cells. Scale bar, 5 μm.

(C) Same as (B) but for U2OS cells.

(D) Droplet formation of PHR-mCherry-HP1α compared with PHR-mCherry in U2OS cells. Expression levels determined by FCS are indicated at the bottom right. Scale bars, 5 μm.

(E) Concentration-dependent droplet formation capacity of PHR-mCherry alone (PHR-mCh) and of fusions of PHR-mCherry with HP1α, the dimerization-deficient mutant HP1α I163A, the PxVxL module of SENP7, FUSN-HP1α and nucleolin (NCL) in U2OS cells. Curves are shown as a guide to the eye. See also Figures S3A and S3B.

(legend continued on next page)

HP1 Represses Transcription but Does Not Form a Droplet When Tethered to Chromatin

To assess whether HP1-HP1 interactions can induce stable droplets at chromatin, we tethered HP1α to an array of *lac*O sites that had been stably integrated into U2OS cells (Janicki et al., 2004). We measured the size and intensity of the recruited assembly around the *lac*O array, using RFP-tagged Tet Repressor (TetR-RFP) bound to the adjacent *tet*O array as a reference (Figure 4A). Tethering GFP-tagged HP1α, NCL, or PML III, a component of PML nuclear bodies, to the *lac*O array resulted in bright nuclear spots (Figure 4B). In contrast to HP1α and NCL, PML III formed a ring-shaped assembly around the *lac*O array, indicating strong additional PML III recruitment via PML-PML interactions adjacent to the *lac*O array. To quantitate the amount of proteins recruited via HP1-HP1, NCL-NCL, and PML-PML interactions, we measured the corresponding intensities at the array in the green channel and normalized it to the TetR intensity at the array (Figure 4C). Using GFP as a control, this analysis revealed how many molecules were recruited to the array in addition to the ones that were directly tethered to *lac*O. For each directly tethered HP1α, NCL, and PML III molecule, 0.6, 1.1, and 13 indirectly bound ones were co-recruited, respectively. To validate the result for HP1α, we conducted a fluorescence recovery after photobleaching (FRAP) analysis of HP1α at the *lac*O array (Figure 4D; see Figure S4 for the control with GFP). From the recovery curves, transient and stable fractions of ~42% and ~58% were obtained, which is in very good agreement with the ratio of directly and indirectly bound HP1α determined from the intensity measurement in Figure 4C. To test the repressive potential of HP1α assemblies at the *lac*O/*tet*O array, we transiently transfected the activator BFP-LacI-VP16 (Rademacher et al., 2017) into cells that stably expressed TetR-PHR-YFP-HP1α. We then measured the transcriptional activity of a reporter located adjacent to the *lac*O/*tet*O sites by qPCR in cells treated with doxycycline to tether HP1α to the *tet*O array, and in cells that were additionally illuminated with blue light to nucleate an HP1α optodroplet at the array. HP1α tethering alone and additional HP1α optodroplet formation efficiently protected the reporter from activation by VP16 (Figure 4E; Figure S4C). Taken together, HP1α has weak capacity to recruit additional HP1α molecules when bound to chromatin, which is lower than self-interactions of NCL and PML III. The response of the reporter indicates that tethered HP1 fusions are functional and that HP1 binding without droplet formation is sufficient for transcriptional repression.

Nucleoli but Not Chromocenters Show Preferential Internal Mixing

Liquid droplets are delimited by a boundary, which can slow down molecular transport at the interface between the droplet and the surrounding phase. This should result in an "internal" pool of molecules that preferentially move within the droplet. We sought to test whether chromocenters contain such an internal HP1 pool that behaves like being confined by a boundary (Figure 5A). To this end, we bleached one half of a chromocenter and measured the fluorescence intensity in the bleached and the non-bleached half (Figure 5B). In the presence of an impermeable boundary that confines molecules to the chromocenter, the intensities in the bleached and non-bleached halves would recover and decay with the same kinetics, respectively. The two signals are linked because recovery in the bleached half would entirely be caused by molecules moving from the non-bleached to the bleached half (Figure 5B, top). In the absence of any boundary that would keep molecules within the chromocenter, the intensity in the bleached half would recover, whereas the intensity in the non-bleached half would only exhibit a subtle and transient intensity drop (Figure 5B, bottom). In this case, the recovering signal would mostly come from regions around the chromocenter. For the intermediate case of a semipermeable boundary, anti-correlated behavior of both halves would be observed while molecules mix internally, until transport across the boundary would lead to recovery of both halves (Figure 5B, center). These scenarios are not dependent on the diffusion coefficient. For rapidly and slowly diffusing molecules, the curves look identical in shape, with the only difference that they are stretched or skewed along the time axis. Accordingly, this experiment provides independent measurements of the permeability of the boundary and of the translational diffusion coefficient, which is advantageous because the latter is affected by several parameters, like local obstacle structure and viscosity (Baum et al., 2014; Digman and Gratton, 2009). To demonstrate the ability of the method to detect boundaries, we transfected iMEF cells with GFP-HP1α and bleached half of the nucleus. The expected anti-correlated behavior between the intensities in the bleached and the non-bleached half described above was observed (Figure 5C). This result reflects the presence of the nuclear membrane, which acts as an impermeable boundary for HP1α. Next we bleached one half of a chromocenter and recorded the intensities over time (Figure 5D). No anti-correlated behavior between intensities in the bleached and the non-bleached half was observed. Rather, the non-bleached half showed a subtle intensity loss and the same recovery kinetics as the bleached half, indicating lack of preferential internal mixing. The same was observed for phosphomimetic variants of HP1 (Figure S5) and for MECP2 (Figure 5E). As a control, we conducted the same experiment with the histone H2B (Figure 5F). The chromatin scaffold in chromocenters did not visibly move on the timescale of 1 min and thus lacked liquid-like behavior as expected for a large polymer on these scales. To compare chromocenters with nucleoli, we transfected cells with GFP-tagged NCL or NPM and bleached one half of a nucleolus. In these experiments, an anti-correlated behavior as expected for partially confined protein pools was observed, with a relatively permeable boundary for NCL (Figure 5G) and a less permeable boundary for NPM (Figure 5H). The fit results for the experiments in Figures 5D–5H are summarized in Table S3. We conclude that nucleoli harbor a pool of proteins that

(F) Stability of droplets containing fusions of PHR-mCherry with the indicated proteins in U2OS cells. Images represent the first frame after blue light exposure (+ blue light) and the time point 8 min after the light pulse (− blue light). NCL and NPM accumulate in nucleoli independently of blue light illumination, which leads to a strong signal in these regions. Error bars represent SEM of at least 10 replicates. Errors for half-lives represent standard fit errors. For RGG_2-HP1 and FUSN-HP1, a subset of droplets persisted, as reflected by the plateaus (dashed lines). Scale bars, 5 μm. See also Figure S3C.

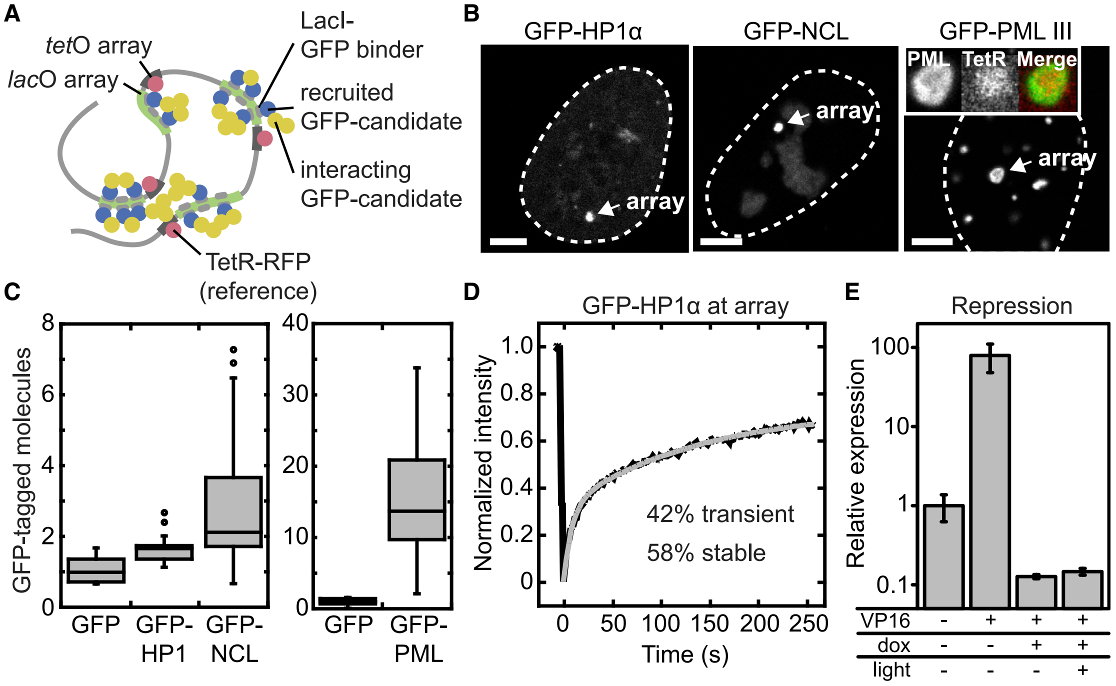

Figure 4. Capacity of HP1α and Other Candidates to Form Droplets When Tethered to Chromatin

(A) Schematic representation of the *lac*O/*tet*O tethering system to test droplet formation.

(B) Confocal microscopy images showing *lac*O arrays bound by the indicated proteins. The inset shows the *tet*O array bound by TetR and the *lac*O array bound by GFP-PML III. Scale bars, 5 μm.

(C) Quantitation of GFP-tagged molecules at *lac*O arrays. Note the different scale for PML III. At least 15 cells were analyzed for each condition.

(D) FRAP analysis of GFP-HP1α at the *lac*O array. From a fit to the data (gray line), a stably (58%) and a transiently (42%) bound fraction of HP1α were resolved. The transient fraction likely represents molecules that accumulate at the array via HP1-HP1 interactions. See also Figures S4A and S4B.

(E) qPCR analysis of the transcriptional activity of the reporter located adjacent to the *lac*O/*tet*O array in cells expressing TetR-PHR-YFP-HP1α and BFP-LacI (mock) or BFP-LacI-VP16 (VP16). For the latter case, cells were treated either only with doxycycline (dox) or with dox and light to assess the repressive potential of HP1α tethering alone compared with HP1α optodroplet formation at the array. Error bars represent SEM from 3 replicates. See also Figure S4C.

preferentially move internally as expected for liquid droplets. This effect was not observed for chromocenters, which appear to be percolated by the nucleoplasm because neither HP1 nor MECP2 experienced any preference for moving inside chromocenters versus moving into the surrounding nucleoplasm.

Chromocenters Exclude Inert Proteins Independent of HP1

It has recently been shown that inert proteins are partially excluded from chromocenters in mouse and *Drosophila* cells (Bancaud et al., 2009; Strom et al., 2017), which has been proposed to be a consequence of LLPS of HP1 (Strom et al., 2017). To test whether exclusion in mouse cells requires HP1, we overexpressed GFP in WT and *Suv39h* dn cells, which lack HP1 enrichment at their chromocenters (Peters et al., 2001; Figure 2). MECP2-RFP was co-transfected as a marker for chromocenters. In agreement with the abovementioned studies, GFP was partially excluded from chromocenters in WT cells (Figure 6A, top). GFP was also partially excluded from chromocenters in *Suv39h* dn cells (Figure 6A, bottom), indicating that H3K9me3 and HP1 enrichment are not responsible for exclusion. To rule out photophysical effects or MECP2-RFP overexpression artifacts, we repeated the experiment with RFP and the chromocenter marker MBD1-GFP (Figure 6B, top) and with GFP in DAPI-stained fixed cells (Figure 6B, bottom), which yielded similar results. We conclude that partial exclusion of GFP/RFP from chromocenters is independent of HP1.

The Liquid Portions of Chromocenters and the Nucleoplasm Have Similar Viscosities

In LLPS, the protein-protein interactions that are responsible for phase separation often lead to increased viscosity of the dense phase (Hyman et al., 2014). An example is the nucleolar protein NPM, which can form droplets that are several hundred times more viscous than water (Feric et al., 2016). Altered viscosity would change the rates of chemical reactions in the two phases. To test whether this is the case for HP1 in living cells, we measured the apparent viscosity experienced by HP1 molecules inside and outside of chromocenters. Because translational diffusion is influenced both by the local viscosity and by the presence of obstacles that can act as diffusion barriers (Baum et al., 2014; Digman and Gratton, 2009), we measured the rotational diffusion of GFP-HP1 (Figure 6C). The latter mainly

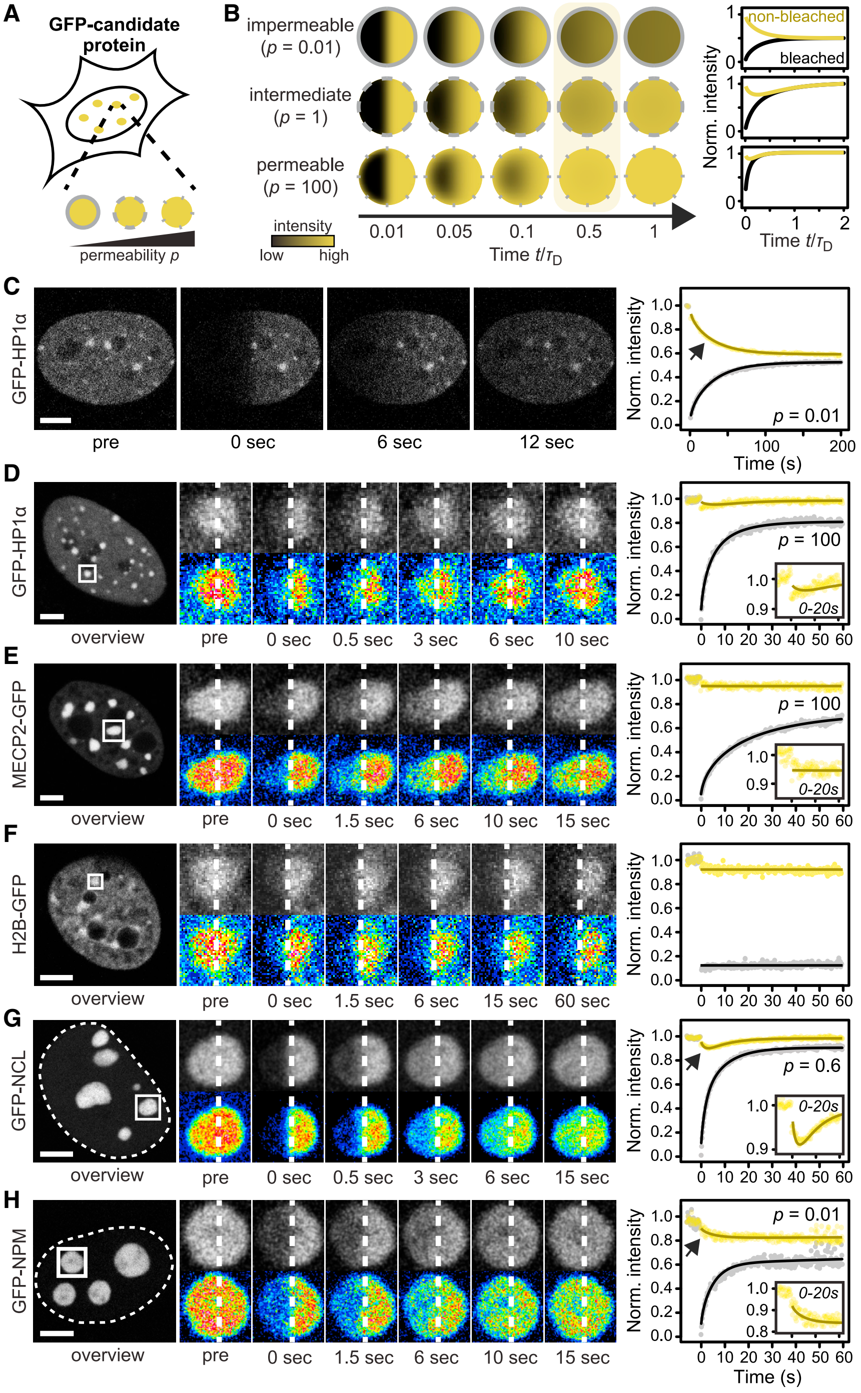

Figure 5. Internal Mixing of Chromocenters and Nucleoli

(A) Schematic representation of the apparent permeability *p* of subcompartments, which is a measure of the prevalence of internal mixing of proteins within the subcompartment in relation to exchange with the surrounding nucleoplasm.

(B) Predicted temporal intensity evolution after having bleached one half of a circle surrounded by a boundary with permeability *p*. The time axis is divided by the diffusion time τ_D, making the plotted curves independent of the diffusion coefficient. At $t/\tau_D = 0.5$ (highlighted time point), the intensity of the non-bleached half is visibly decreased if a boundary is present.

(C) Half-nucleus bleach for cells expressing GFP-HP1α (n = 5). The arrow points to the intensity decrease in the non-bleached half that reflects preferential internal mixing.

(D) Half-chromocenter bleach for cells expressing GFP-HP1α (n = 35). The inset shows the intensity of the non-bleached half during the first 20 s of the experiment. Magnified images were smoothed for clarity. See also Figure S5.

(E) Same as (D) but for MECP2-GFP (n = 22).

(F) Same as (D) but for H2B-GFP (n = 19).

(G) Half-nucleolus bleach for cells expressing GFP-NCL (n = 28). The inset shows the intensity of the non-bleached half during the first 20 s of the experiment. The arrow points to the intensity decrease in the non-bleached half that reflects preferential internal mixing.

(H) Same as (G) but for GFP-NPM (n = 14).

Scale bars in (C)–(H), 5 μm.

depends on the local viscosity because it occurs on shorter timescales, where binding interactions with chromatin and collisions with diffusion barriers become negligible (Baum et al., 2014; Oura et al., 2016; Verkman, 2002). Rotational diffusion of HP1 should be sensitive to any interactions among HP1 molecules that depend on their relative orientation; e.g., dipole interactions potentially driving LLPS (Brangwynne et al., 2015). We used polarization-sensitive fluorescence correlation spectroscopy (Pol-FCS) with two detector pairs to record the fluorescence signal parallel and perpendicular to the excitation laser beam to resolve translational and rotational diffusion (Figures 6D and 6E; STAR Methods). Fitting the correlation curves in Figure 6E yielded similar rotational correlation times of GFP-HP1 in the nucleoplasm (τ_r = 117 ± 9 ns) and in chromocenters (τ_r = 111 ± 8 ns) (Figure 6F; Table S4). In the cytoplasm, however, GFP-HP1 rotated faster than in the nucleus (τ_r = 74 ± 7 ns), which might reflect a reduced size of the rotating HP1 species because of decreased HP1 dimerization or lack of nuclear binding partners. As a reference, we conducted Pol-FCS measurements with purified GFP-HP1 at a concentration of ~50 nM; i.e., below the concentration for dimerization or self-association, in glycerol-water mixtures with different viscosities (Figure 6G). As expected, the fitted rotational diffusion times increased with the glycerol concentration and viscosity of the mixtures (Figure 6H; Figure S6; Table S5), demonstrating that Pol-FCS is suited to measure the viscosity of HP1 solutions. Taken together, these experiments show that the apparent viscosity experienced by GFP-HP1 inside and outside of chromocenters is similar. Thus, HP1 proteins

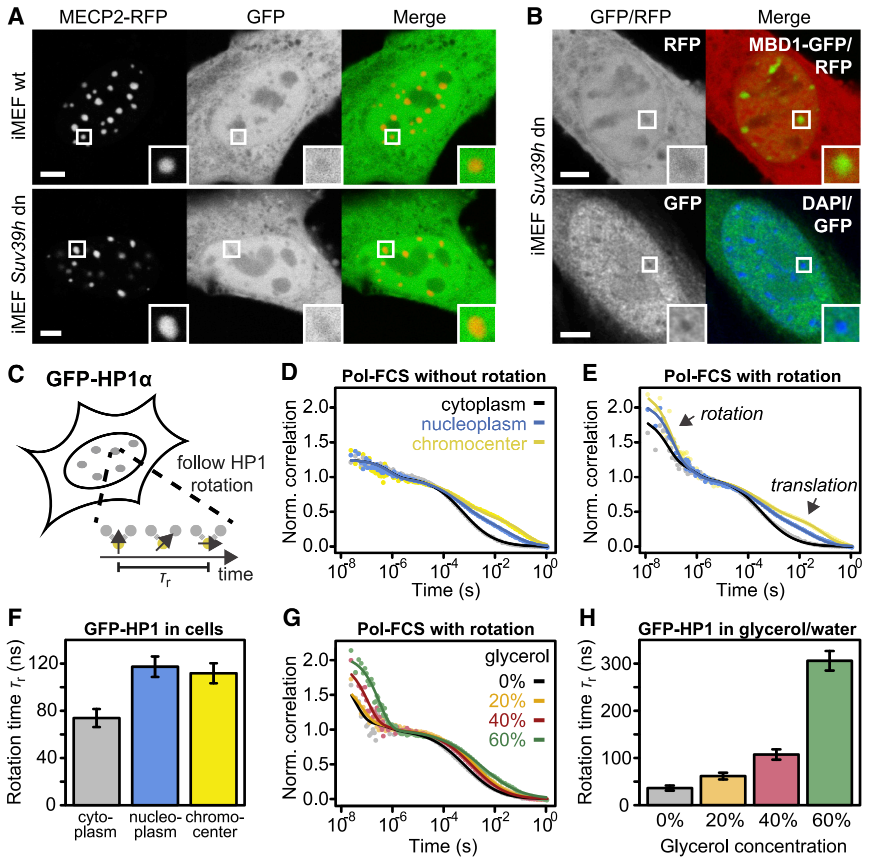

Figure 6. Accessibility and Local Viscosity of Chromocenters

(A) Representative confocal images of WT and *Suv39h* dn cells expressing GFP and MECP2-RFP. Merge images: red, MECP2-RFP; green, GFP. Insets show magnified chromocenters with partial GFP exclusion. Scale bars, 5 μm.

(B) Same as (A) but for *Suv39h* dn cells expressing RFP and MBD1-GFP (top) and for fixed and DAPI-stained *Suv39h* dn cells expressing GFP (bottom).

(C) Schematic representation of the polarization-sensitive fluorescence correlation spectroscopy (Pol-FCS) experiment. Pol-FCS measures the local viscosity of chromocenters via rotational diffusion of GFP-HP1. HP1-HP1 interactions within a dense liquid phase formed by LLPS are expected to increase local viscosity.

(D) Pol-FCS measurement of GFP-HP1 in living cells with crossed detectors to resolve only translational diffusion (n = 19).

(E) Pol-FCS measurement of GFP-HP1 in living cells with parallel detectors to resolve both translational and rotational diffusion (n = 19; data for the detector configurations in this and in D were acquired in the same measurements).

(F) Rotational diffusion times obtained from a fit to the Pol-FCS data shown in (E). Error bars represent standard fit errors. See also Table S4.

(G) Pol-FCS measurement with parallel detectors of GFP-HP1 in glycerol/water mixtures with the indicated glycerol concentrations.

(H) Rotational diffusion times obtained from fitting the Pol-FCS measurements in (G). Error bars represent standard fit errors. See also Figure S6 and Table S5.

that are not bound to chromatin do not experience detectable directional HP1-HP1 interactions, which would be a hallmark of LLPS. Rather, our results suggest that chromocenters are percolated by the same liquid as the surrounding chromatin.

The Size but Not the HP1 Level of Chromocenters Is Buffered

Another hallmark feature of liquid droplets is concentration buffering (Banani et al., 2017). It refers to the effect that the total volume of all droplets in the cell scales with the cellular concentration of the phase-separating protein, whereas its concentrations inside and outside of droplets remain constant (Figure 7A). A collapsed chromatin globule, however, behaves differently. It remains constant in size while the internal protein concentration changes. To test these predictions, we transfected iMEF cells with a plasmid encoding untagged HP1α and the fluorescent marker ZsGreen, separated by an internal ribosomal entry site (IRES). After immunostaining of HP1α, we imaged the cells and grouped them into three categories with low, medium, and high expression levels of ZsGreen (Figure S7A; Figure 7B). We segmented chromocenters based on the DAPI channel and measured HP1 and DAPI signals as well as the image area covered by chromocenters in each of the three groups (Figure 7C). HP1α signals in the nucleoplasm and chromocenters increased with ZsGreen levels, whereas the chromocenter area and DAPI signal remained constant. This behavior is fully consistent with the predictions for a collapsed chromatin globule but not with a liquid HP1 droplet.

Activation of Chromocenters Triggers a Sharp Transition to a Decondensed State

Next we tested how chromocenters respond to an activator and whether HP1 affects their response. We transfected iMEF cells with plasmids coding for the GFP-tagged strong activator dCas9-VPR (Chavez et al., 2015) and a guide RNA that targets the major satellite repeats located in chromocenters (Figure 7D). As a control, we used GFP-tagged dCas9 lacking VPR (dCas9-mock). After fixation, we visualized acetylation of histone H3 at lysine 27 (H3K27ac) by immunostaining and DNA by DAPI staining and grouped the cells into three categories with low, medium, and high dCas9 signals at satellite repeats (Figure S7B). Recruitment of dCas9-VPR induced decondensation of chromocenters (Figure 7E) and an increase of H3K27ac at major satellite repeats (Figures S7C and S7D). Cells showed a spotty distribution of dCas9-VPR-bound satellite repeats, suggesting that chromocenters contain substructures that decondense individually. To quantitatively assess how decondensation occurs as a function of dCas9-VPR binding, we measured DAPI and H3K27ac levels at dCas9-bound major satellites and the image area covered by major satellites as a function of dCas9-VPR binding (Figures 7F and 7G; Figure S7C). A steep increase of the satellite area was already apparent for low dCas9-VPR levels, followed by a

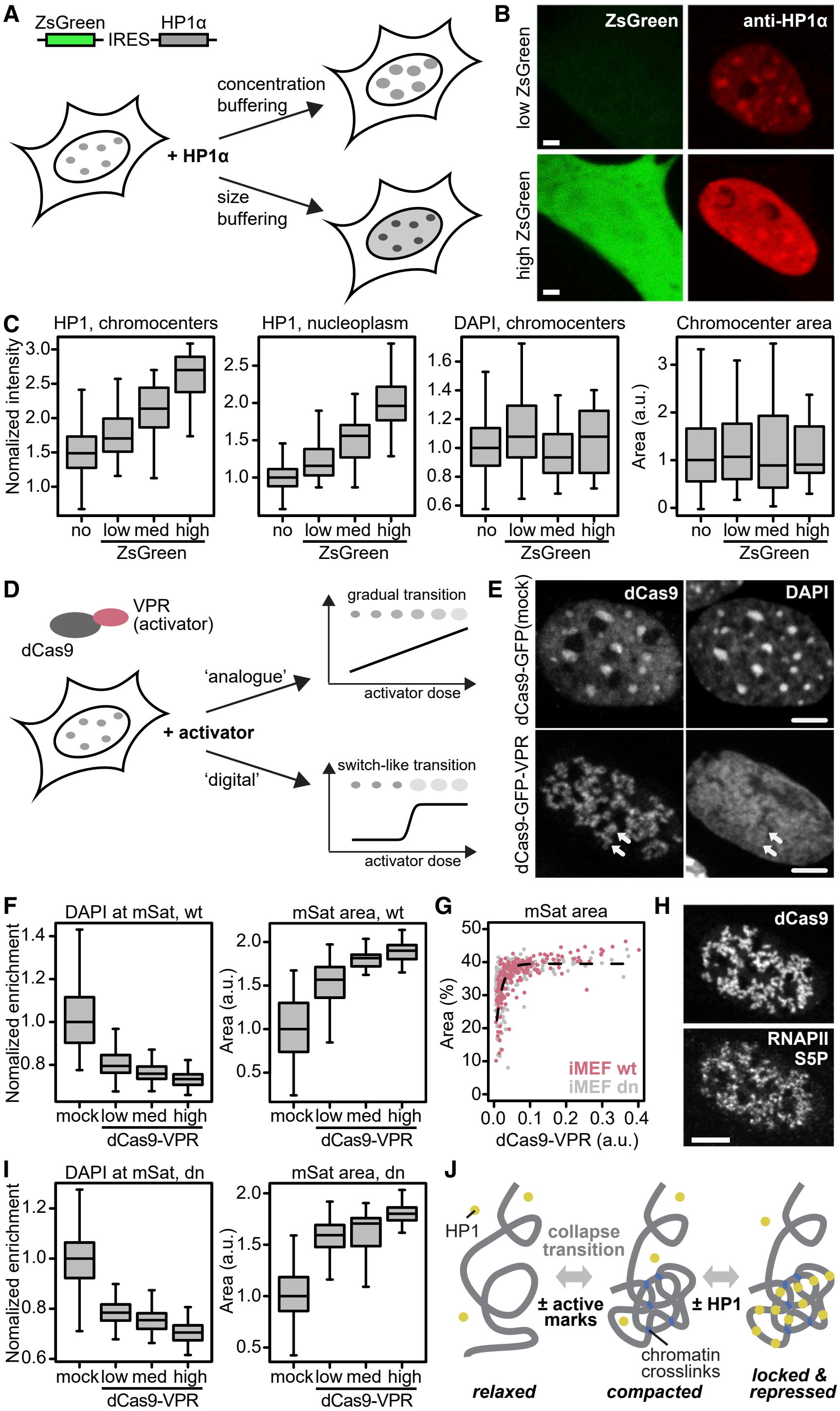

Figure 7. Regulation of Chromocenter Size and Compaction

(A) Schematic representation of the plasmid used for HP1α overexpression and model predictions. Concentration buffering: HP1α overexpression increases chromocenter sizes, whereas HP1α levels inside and outside of chromocenters remain constant. Size buffering: chromocenters retain their size, whereas HP1α levels increase inside and outside of chromocenters.

(B) Representative confocal images of cells expressing low (top) or high (bottom) levels of ZsGreen and HP1α. Scale bars, 5 μm.

(C) HP1α levels inside and outside of chromocenters, DAPI levels at chromocenters, and chromocenter area as a function of HP1α overexpression. The groups with no, low, medium, and high ZsGreen levels contained 1406, 45, 24, and 13 cells, respectively. See also Figure S7A.

(D) Schematic representation of the epigenetic editing experiment to study heterochromatin decondensation. A switch-like dose response with digital compaction states is indicative of a collapse transition.

(E) Representative confocal images of cells with dCas9-GFP-VPR or dCas9-GFP (mock) bound to major satellite repeats (mSats). The white arrows highlight decondensed chromocenter structures. Scale bars, 5 μm. See also Figure S7D.

(F) DAPI levels at chromocenters and chromocenter area as a function of dCas9 levels at major satellites in WT iMEF cells. The dCas9-mock group contained 155 cells; the groups with low, medium, and high dCas9-VPR levels contained 83, 42, and 83 cells, respectively. See also Figures S7B and S7C.

(G) Relationship between dCas9-VPR recruitment and chromocenter decondensation in WT iMEF (red) and *Suv39h* dn iMEF (gray) cells. The dashed line represents a fit with an exponential functional (as a guide to the eye). See also Figure S7F.

(H) Representative confocal images of dCas9-VPR-expressing cells with major satellites enriched for RNA polymerase II phosphorylated at serine 5 (Pol II S5P). Scale bar, 5 μm. See also Figure S7E.

(I) Same as (F) but for *Suv39h* dn iMEF cells. The dCas9-mock group contained 148 cells; the groups with low, medium, and high dCas9-VPR levels contained 95, 48, and 95 cells, respectively. See also Figures S7B and S7C.

(J) Proposed model for heterochromatin separation that recapitulates our data. The left and the right state correspond to the endogenous eu- and heterochromatin states, respectively.

plateau for medium and high dCas9-VPR levels. This behavior is indicative of a phase transition between a compacted and a relaxed state, as expected for a collapsed globule that loses interactions among its segments (Kim and Han, 2010; Leibler, 1980). Decondensed satellite repeats were enriched for RNA polymerase II phosphorylated at serine 5 (Figure 7H; Figure S7E), suggesting that the relaxation induced by dCas9-VPR was associated with deprotection and transcriptional activation. *Suv39h* dn and WT cells reacted similarly to dCas9-VPR and dCas9-GFP (Figures 7G and 7I; Figures S7C–S7F). These results suggest that heterochromatin compaction is digital; it adopts two distinct states that are buffered in the sense that the natural condensed state tolerates loss of H3K9me3 and HP1, whereas the relaxed state tolerates further accumulation of activators

without showing further decondensation. The transition between both states is largely independent of HP1 and H3K9me3, which might be responsible for transcriptional repression in the compacted state (Figure 7J).

DISCUSSION

In this study, we assessed key biophysical properties of mouse pericentric heterochromatin that are relevant for understanding chromatin compartmentalization and its consequences for the accessibility, spreading, and maintenance of heterochromatin. To this end, we combined complementary techniques, some of which involved fluorescently tagged HP1. Several crucial features of endogenous HP1α are preserved for GFP-HP1α; e.g., its ability to bind to chromocenters, to undergo protein-protein interactions via its chromo- and chromoshadow domain (Müller-Ott et al., 2014; Romeo et al., 2015; Thiru et al., 2004), to form DNA-induced droplets *in vitro* (Figure 1), and to repress a transcriptional reporter (Figure 4). To address potential differences between HP1α fusions and the endogenous proteins, we also studied untagged HP1α where possible (e.g., Figure 7), employed a tagged HP1-interacting PxVxL motif instead of tagged HP1 (Figure 3), and compared GFP-HP1α with MECP2-GFP, another key heterochromatin protein (e.g., Figure 5). Combining our results led us to the following conclusions. (1) HP1α forms stable droplets *in vitro* at high concentrations and when mixed with DNA, but neither forms stable droplets in the nucleoplasm nor when tethered to chromatin in living cells. This result suggests that heterochromatin maintenance is independent from liquid droplet formation of HP1α. (2) Chromocenters lack preferential internal mixing and have the same viscosity as the surrounding euchromatin, indicating that both types of chromatin are percolated by the same nucleoplasmic liquid and are accessible to factors dissolved in it. (3) Partial exclusion of GFP from chromocenters is independent of HP1. Thus, access to chromocenters by an inert tracer protein is not regulated by HP1 but likely by the tracer's ability to penetrate the denser chromocenter structure. (4) The HP1α level in chromocenters, but not the size of chromocenters, follows the total cellular HP1α level, indicating that heterochromatin spreading is not directly linked to the HP1α concentration as would be expected for liquid droplets. However, it is conceivable that spreading of the repressive heterochromatin state (on smaller scales than assessed here) is linked to HP1 by other means because HP1 is a central heterochromatin protein with many interaction partners and functions. (5) Compaction of chromocenters is sensitive to the presence of a strong activator but not to HP1. Upon forced activation, heterochromatin compaction shows a switch-like transition, and chromocenters abruptly decompact, indicating that compaction is digital, as expected for the formation of a collapsed polymer globule.

Taken together, we report that the global compaction, accessibility, and size of chromocenters is largely independent of HP1. This result is in line with a number of earlier studies showing that compact chromocenters and other heterochromatin domains can form and be maintained without HP1 binding (e.g., Gilbert et al., 2003; Mateos-Langerak et al., 2007; Peters et al., 2001; Schotta et al., 2004). We find that chromocenters show hallmarks of collapsed chromatin globules rather than liquid droplets, as judged from their permeability to the liquid portion of the nucleoplasm and their dose response to transcriptional activators. Accordingly, heterochromatin is expected to be accessible to nucleoplasmic factors that are able to penetrate the more compact collapsed state. Thus, separation between eu- and heterochromatin relies on cues that control the collapse. The collapsed and the relaxed state tolerate moderate perturbations without changing their compaction, which leads us to propose that these digital states represent the two fundamental modes of chromatin compaction. We speculate that the transition between the two states is driven by a combination of heterochromatin-specific bridging interactions and the intrinsic property of pericentric repeats to self-associate, possibly because of their particular sequence properties and increased nucleosome density (Kang et al., 2018; Pepenella et al., 2014). Heterochromatin might decondense into the relaxed state when at least one of these contributions is lost. This process can be triggered by local recruitment of activators or by global inhibition of histone deacetylases (Taddei et al., 2001), which creates a hyperacetylated chromatin state with reduced chromatin bridging (Eberharter and Becker, 2002). On a genome-wide scale, distinct separation of both chromatin compaction states by a collapse transition might be linked to segregation of chromatin into the A/B compartments that have been observed in contact matrices acquired by Hi-C early on (Lieberman-Aiden et al., 2009). The role of HP1 might be to participate in heterochromatin-specific bridging and to protect heterochromatin against spurious induction of satellite repeat transcription by activators that are weaker and/or bind more sparsely to chromocenters than dCas9-VPR. In this manner, HP1 would stabilize the silenced collapsed heterochromatin state without being sufficient to reestablish it when it is lost.

Our work presented here sheds light on the biophysical basis of chromatin compartmentalization and the consequences arising from it. We anticipate that it will help dissect the contributions of protein self-association, liquid phase separation, and polymer collapse to the structure and function of chromatin subcompartments in different systems to uncover the general rules governing chromatin partitioning across cell types and species.

STAR★METHODS

Detailed methods are provided in the online version of this paper and include the following:

- KEY RESOURCES TABLE
- LEAD CONTACT AND MATERIALS AVAILABILITY
- EXPERIMENTAL MODEL AND SUBJECT DETAILS
- METHOD DETAILS
    - Plasmids
    - Protein expression, *in vitro* droplet formation and Western Blotting
    - Immunostaining and transfection
    - Confocal and STED microscopy of living and fixed cells
    - Optodroplet induction and stability measurements

- ○ Protein mobility measurements
- ○ Measurement of reporter activity by real-time quantitative PCR
- ● QUANTIFICATION AND STATISTICAL ANALYSIS
  - ○ Turbidity analysis
  - ○ Image correlation spectroscopy
  - ○ Quantification of optodroplet abundance and lifetime
  - ○ FRAP analysis of HP1 at lacO-array
  - ○ FRAP analysis of half-bleached cellular structures
  - ○ Pol-FCS analysis
  - ○ Intensity analysis of confocal images for dose-response relationships
- ● DATA AND CODE AVAILABILITY

SUPPLEMENTAL INFORMATION

Supplemental Information can be found online at https://doi.org/10.1016/j.molcel.2020.02.005.

ACKNOWLEDGMENTS

We thank Clara-Marie Gürth, Alina Batzilla, and Inn Chung for support with the STED work and Corentin Moevus, Olivier Cuvier, Angela Taddei, Olivier Gadal, Daniel Jost, and Kerstin Bystricky for helpful comments and discussions. We thank the light microscopy facilities at DKFZ Heidelberg and CBI Toulouse for help. This work was supported by DFG grant RI1283/16-1 (to K.R.), grants from the Netherlands Organization for Scientific Research (680-50-1501) and the European Molecular Biology Organization (ALTF 1516-2015) (to R.V.), DFG grant HE4559/6-1 (PhotoQuant) (to D.-P.H.), and a grant from the European Research Council (ERC) under the European Union Horizon 2020 Research and Innovation Program (grant agreement 804023 to F.E.).

AUTHOR CONTRIBUTIONS

Acquisition of Data, F.E., A.R., R.V., J.T., L.F., R.W., E.S., K.Y., J.H., C.B., S.S., C.N., and A.A.A.; Analysis of Data, F.E., A.R., R.V., J.T., L.F., R.W., E.S., K.Y., and J.H.; Drafting of the Manuscript, F.E. and K.R.; Reviewing of the Manuscript, F.E., A.R., R.V., J.T., L.F., K.Y., J.H., A.A.A., D.-P.H., and K.R.; Supervision, F.E., J.E., D.-P.H., and K.R.; Study Design and Coordination, F.E. and K.R.

DECLARATION OF INTERESTS

The authors declare no competing interests.

Received: August 7, 2019
Revised: December 20, 2019
Accepted: February 4, 2020
Published: February 25, 2020

SUPPORTING CITATIONS

The following references appear in the Supplemental Information: Casas-Delucchi et al. (2012); Cheng (2008); Lavalette et al. (1999); Weber and Brangwynne (2015).

REFERENCES

Banani, S.F., Lee, H.O., Hyman, A.A., and Rosen, M.K. (2017). Biomolecular condensates: organizers of cellular biochemistry. Nat. Rev. Mol. Cell Biol. *18*, 285–298.

Bancaud, A., Huet, S., Daigle, N., Mozziconacci, J., Beaudouin, J., and Ellenberg, J. (2009). Molecular crowding affects diffusion and binding of nuclear proteins in heterochromatin and reveals the fractal organization of chromatin. EMBO J. *28*, 3785–3798.

Bannister, A.J., Zegerman, P., Partridge, J.F., Miska, E.A., Thomas, J.O., Allshire, R.C., and Kouzarides, T. (2001). Selective recognition of methylated lysine 9 on histone H3 by the HP1 chromo domain. Nature *410*, 120–124.

Baum, M., Erdel, F., Wachsmuth, M., and Rippe, K. (2014). Retrieving the intracellular topology from multi-scale protein mobility mapping in living cells. Nat. Commun. *5*, 4494.

Bracha, D., Walls, M.T., Wei, M.T., Zhu, L., Kurian, M., Avalos, J.L., Toettcher, J.E., and Brangwynne, C.P. (2018). Mapping Local and Global Liquid Phase Behavior in Living Cells Using Photo-Oligomerizable Seeds. Cell *175*, 1467–1480.e13.

Brangwynne, C.P., Tompa, P., and Pappu, R.V. (2015). Polymer physics of intracellular phase transitions. Nat. Phys. *11*, 899–904.

Carslaw, H.S., and Jaeger, J.C. (1959). Conduction of Heat in Solids, Second Edition (Oxford: Oxford University Press).

Casas-Delucchi, C.S., van Bemmel, J.G., Haase, S., Herce, H.D., Nowak, D., Meilinger, D., Stear, J.H., Leonhardt, H., and Cardoso, M.C. (2012). Histone hypoacetylation is required to maintain late replication timing of constitutive heterochromatin. Nucleic Acids Res. *40*, 159–169.

Caudron-Herger, M., Pankert, T., Seiler, J., Németh, A., Voit, R., Grummt, I., and Rippe, K. (2015). Alu element-containing RNAs maintain nucleolar structure and function. EMBO J. *34*, 2758–2774.

Cavalli, G., and Misteli, T. (2013). Functional implications of genome topology. Nat. Struct. Mol. Biol. *20*, 290–299.

Chavez, A., Scheiman, J., Vora, S., Pruitt, B.W., Tuttle, M., P R Iyer, E., Lin, S., Kiani, S., Guzman, C.D., Wiegand, D.J., et al. (2015). Highly efficient Cas9-mediated transcriptional programming. Nat. Methods *12*, 326–328.

Cheng, N.S. (2008). Formula for the viscosity of a glycerol-water mixture. Ind. Eng. Chem. Res. *47*, 3285–3288.

Cook, P.R., and Marenduzzo, D. (2018). Transcription-driven genome organization: a model for chromosome structure and the regulation of gene expression tested through simulations. Nucleic Acids Res. *46*, 9895–9906.

Digman, M.A., and Gratton, E. (2009). Imaging barriers to diffusion by pair correlation functions. Biophys. J. *97*, 665–673.

Eberharter, A., and Becker, P.B. (2002). Histone acetylation: a switch between repressive and permissive chromatin. Second in review series on chromatin dynamics. EMBO Rep. *3*, 224–229.

Elgin, S.C., and Reuter, G. (2013). Position-effect variegation, heterochromatin formation, and gene silencing in Drosophila. Cold Spring Harb. Perspect. Biol. *5*, a017780.

Emmott, E., and Hiscox, J.A. (2009). Nucleolar targeting: the hub of the matter. EMBO Rep. *10*, 231–238.

Erdel, F., and Rippe, K. (2018). Formation of Chromatin Subcompartments by Phase Separation. Biophys. J. *114*, 2262–2270.

Feric, M., Vaidya, N., Harmon, T.S., Mitrea, D.M., Zhu, L., Richardson, T.M., Kriwacki, R.W., Pappu, R.V., and Brangwynne, C.P. (2016). Coexisting Liquid Phases Underlie Nucleolar Subcompartments. Cell *165*, 1686–1697.

Fodor, B.D., Shukeir, N., Reuter, G., and Jenuwein, T. (2010). Mammalian Su(var) genes in chromatin control. Annu. Rev. Cell Dev. Biol. *26*, 471–501.

Gilbert, N., Boyle, S., Sutherland, H., de Las Heras, J., Allan, J., Jenuwein, T., and Bickmore, W.A. (2003). Formation of facultative heterochromatin in the absence of HP1. EMBO J. *22*, 5540–5550.

Gorlitz, F., Hoyer, P., Falk, H., Kastrup, L., Engelhardt, J., and Hell, S.W. (2014). A STED microscope designed for routine biomedical applications. Prog. Electromagnetics Res. *147*, 57–68.

Grußmayer, K.S., and Herten, D.P. (2017). Time-resolved molecule counting by photon statistics across the visible spectrum. Phys. Chem. Chem. Phys. *19*, 8962–8969.

Hiragami-Hamada, K., Soeroes, S., Nikolov, M., Wilkins, B., Kreuz, S., Chen, C., De La Rosa-Velázquez, I.A., Zenn, H.M., Kost, N., Pohl, W., et al. (2016). Dynamic and flexible H3K9me3 bridging via HP1β dimerization establishes a plastic state of condensed chromatin. Nat. Commun. *7*, 11310.

Hyman, A.A., Weber, C.A., and Jülicher, F. (2014). Liquid-liquid phase separation in biology. Annu. Rev. Cell Dev. Biol. *30*, 39–58.

Janicki, S.M., Tsukamoto, T., Salghetti, S.E., Tansey, W.P., Sachidanandam, R., Prasanth, K.V., Ried, T., Shav-Tal, Y., Bertrand, E., Singer, R.H., and Spector, D.L. (2004). From silencing to gene expression: real-time analysis in single cells. Cell *116*, 683–698.

Jegou, T., Chung, I., Heuvelman, G., Wachsmuth, M., Görisch, S.M., Greulich-Bode, K.M., Boukamp, P., Lichter, P., and Rippe, K. (2009). Dynamics of telomeres and promyelocytic leukemia nuclear bodies in a telomerase-negative human cell line. Mol. Biol. Cell *20*, 2070–2082.

Jost, D., Vaillant, C., and Meister, P. (2017). Coupling 1D modifications and 3D nuclear organization: data, models and function. Curr. Opin. Cell Biol. *44*, 20–27.

Kang, H., Yoo, J., Sohn, B.K., Lee, S.W., Lee, H.S., Ma, W., Kee, J.M., Aksimentiev, A., and Kim, H. (2018). Sequence-dependent DNA condensation as a driving force of DNA phase separation. Nucleic Acids Res. *46*, 9401–9413.

Kennedy, M.J., Hughes, R.M., Peteya, L.A., Schwartz, J.W., Ehlers, M.D., and Tucker, C.L. (2010). Rapid blue-light-mediated induction of protein interactions in living cells. Nat. Methods *7*, 973–975.

Kilic, S., Felekyan, S., Doroshenko, O., Boichenko, I., Dimura, M., Vardanyan, H., Bryan, L.C., Arya, G., Seidel, C.A.M., and Fierz, B. (2018). Single-molecule FRET reveals multiscale chromatin dynamics modulated by HP1α. Nat. Commun. *9*, 235.

Kim, J.K., and Han, C.D. (2010). Phase Behavior and Phase Transitions in AB- and ABA-type Microphase-Separated Block Copolymers. Adv. Polym. Sci. *231*, 77–145.

Kundaje, A., Meuleman, W., Ernst, J., Bilenky, M., Yen, A., Heravi-Moussavi, A., Kheradpour, P., Zhang, Z., Wang, J., Ziller, M.J., et al.; Roadmap Epigenomics Consortium (2015). Integrative analysis of 111 reference human epigenomes. Nature *518*, 317–330.

Larson, A.G., Elnatan, D., Keenen, M.M., Trnka, M.J., Johnston, J.B., Burlingame, A.L., Agard, D.A., Redding, S., and Narlikar, G.J. (2017). Liquid droplet formation by HP1α suggests a role for phase separation in heterochromatin. Nature *547*, 236–240.

Lavalette, D., Tétreau, C., Tourbez, M., and Blouquit, Y. (1999). Microscopic viscosity and rotational diffusion of proteins in a macromolecular environment. Biophys. J. *76*, 2744–2751.

Leibler, L. (1980). Theory of Microphase Separation in Block Co-Polymers. Macromolecules *13*, 1602–1617.

Lieberman-Aiden, E., van Berkum, N.L., Williams, L., Imakaev, M., Ragoczy, T., Telling, A., Amit, I., Lajoie, B.R., Sabo, P.J., Dorschner, M.O., et al. (2009). Comprehensive mapping of long-range interactions reveals folding principles of the human genome. Science *326*, 289–293.

Machida, S., Takizawa, Y., Ishimaru, M., Sugita, Y., Sekine, S., Nakayama, J.I., Wolf, M., and Kurumizaka, H. (2018). Structural Basis of Heterochromatin Formation by Human HP1. Mol. Cell *69*, 385–397.e8.

MacPherson, Q., Beltran, B., and Spakowitz, A.J. (2018). Bottom-up modeling of chromatin segregation due to epigenetic modifications. Proc. Natl. Acad. Sci. USA *115*, 12739–12744.

Mateos-Langerak, J., Brink, M.C., Luijsterburg, M.S., van der Kraan, I., van Driel, R., and Verschure, P.J. (2007). Pericentromeric heterochromatin domains are maintained without accumulation of HP1. Mol. Biol. Cell *18*, 1464–1471.

Michieletto, D., Orlandini, E., and Marenduzzo, D. (2016). Polymer model with Epigenetic Recoloring Reveals a Pathway for the de novo Establishment and 3D Organization of Chromatin Domains. Phys. Rev. X *6*, 41047.

Müller, K.P., Erdel, F., Caudron-Herger, M., Marth, C., Fodor, B.D., Richter, M., Scaranaro, M., Beaudouin, J., Wachsmuth, M., and Rippe, K. (2009). Multiscale analysis of dynamics and interactions of heterochromatin protein 1 by fluorescence fluctuation microscopy. Biophys. J. *97*, 2876–2885.

Müller-Ott, K., Erdel, F., Matveeva, A., Mallm, J.-P., Rademacher, A., Hahn, M., Bauer, C., Zhang, Q., Kaltofen, S., Schotta, G., et al. (2014). Specificity, propagation, and memory of pericentric heterochromatin. Mol. Syst. Biol. *10*, 746.

Nicodemi, M., and Pombo, A. (2014). Models of chromosome structure. Curr. Opin. Cell Biol. *28*, 90–95.

Nuebler, J., Fudenberg, G., Imakaev, M., Abdennur, N., and Mirny, L.A. (2018). Chromatin organization by an interplay of loop extrusion and compartmental segregation. Proc. Natl. Acad. Sci. USA *115*, E6697–E6706.

Otsu, N. (1979). A Threshold Selection Method from Gray-Level Histograms. IEEE Transactions on Systems, Man, and Cybernetics *9*, 62–66.

Oura, M., Yamamoto, J., Ishikawa, H., Mikuni, S., Fukushima, R., and Kinjo, M. (2016). Polarization-dependent fluorescence correlation spectroscopy for studying structural properties of proteins in living cell. Sci. Rep. *6*, 31091.

Pau, G., Fuchs, F., Sklyar, O., Boutros, M., and Huber, W. (2010). EBImage–an R package for image processing with applications to cellular phenotypes. Bioinformatics *26*, 979–981.

Pepenella, S., Murphy, K.J., and Hayes, J.J. (2014). Intra- and inter-nucleosome interactions of the core histone tail domains in higher-order chromatin structure. Chromosoma *123*, 3–13.

Peters, A.H., O'Carroll, D., Scherthan, H., Mechtler, K., Sauer, S., Schöfer, C., Weipoltshammer, K., Pagani, M., Lachner, M., Kohlmaier, A., et al. (2001). Loss of the Suv39h histone methyltransferases impairs mammalian heterochromatin and genome stability. Cell *107*, 323–337.

Petersen, N.O., Höddelius, P.L., Wiseman, P.W., Seger, O., and Magnusson, K.E. (1993). Quantitation of membrane receptor distributions by image correlation spectroscopy: concept and application. Biophys. J. *65*, 1135–1146.

Probst, A.V., and Almouzni, G. (2008). Pericentric heterochromatin: dynamic organization during early development in mammals. Differentiation *76*, 15–23.

R Core Team (2017). R: A language and environment for statistical computing (R Foundation for Statistical Computing).

Rademacher, A., Erdel, F., Trojanowski, J., Schumacher, S., and Rippe, K. (2017). Real-time observation of light-controlled transcription in living cells. J. Cell Sci. *130*, 4213–4224.

Romeo, K., Louault, Y., Cantaloube, S., Loiodice, I., Almouzni, G., and Quivy, J.P. (2015). The SENP7 SUMO-Protease Presents a Module of Two HP1 Interaction Motifs that Locks HP1 Protein at Pericentric Heterochromatin. Cell Rep. *10*, 771–782.

Rothbauer, U., Zolghadr, K., Muyldermans, S., Schepers, A., Cardoso, M.C., and Leonhardt, H. (2008). A versatile nanotrap for biochemical and functional studies with fluorescent fusion proteins. Mol. Cell. Proteomics *7*, 282–289.

Schotta, G., Lachner, M., Sarma, K., Ebert, A., Sengupta, R., Reuter, G., Reinberg, D., and Jenuwein, T. (2004). A silencing pathway to induce H3-K9 and H4-K20 trimethylation at constitutive heterochromatin. Genes Dev. *18*, 1251–1262.

Schuster, B.S., Reed, E.H., Parthasarathy, R., Jahnke, C.N., Caldwell, R.M., Bermudez, J.G., Ramage, H., Good, M.C., and Hammer, D.A. (2018). Controllable protein phase separation and modular recruitment to form responsive membraneless organelles. Nat. Commun. *9*, 2985.

Shin, Y., Berry, J., Pannucci, N., Haataja, M.P., Toettcher, J.E., and Brangwynne, C.P. (2017). Spatiotemporal Control of Intracellular Phase Transitions Using Light-Activated optoDroplets. Cell *168*, 159–171.e14.

Strom, A.R., Emelyanov, A.V., Mir, M., Fyodorov, D.V., Darzacq, X., and Karpen, G.H. (2017). Phase separation drives heterochromatin domain formation. Nature *547*, 241–245.

Taddei, A., Maison, C., Roche, D., and Almouzni, G. (2001). Reversible disruption of pericentric heterochromatin and centromere function by inhibiting deacetylases. Nat. Cell Biol. *3*, 114–120.

Taylor, N.O., Wei, M.T., Stone, H.A., and Brangwynne, C.P. (2019). Quantifying Dynamics in Phase-Separated Condensates Using Fluorescence Recovery after Photobleaching. Biophys. J. *117*, 1285–1300.

Thiru, A., Nietlispach, D., Mott, H.R., Okuwaki, M., Lyon, D., Nielsen, P.R., Hirshberg, M., Verreault, A., Murzina, N.V., and Laue, E.D. (2004). Structural

basis of HP1/PXVXL motif peptide interactions and HP1 localisation to heterochromatin. EMBO J. *23*, 489–499.

van Steensel, B., and Furlong, E.E.M. (2019). The role of transcription in shaping the spatial organization of the genome. Nat. Rev. Mol. Cell Biol. *20*, 327–337.

Verkman, A.S. (2002). Solute and macromolecule diffusion in cellular aqueous compartments. Trends Biochem. Sci. *27*, 27–33.

Wang, L., Gao, Y., Zheng, X., Liu, C., Dong, S., Li, R., Zhang, G., Wei, Y., Qu, H., Li, Y., et al. (2019). Histone Modifications Regulate Chromatin Compartmentalization by Contributing to a Phase Separation Mechanism. Mol. Cell *76*, 646–659.e6.

Weber, S.C., and Brangwynne, C.P. (2015). Inverse size scaling of the nucleolus by a concentration-dependent phase transition. Curr. Biol. *25*, 641–646.

Zhang, Y.J., Guo, L., Gonzales, P.K., Gendron, T.F., Wu, Y., Jansen-West, K., O'Raw, A.D., Pickles, S.R., Prudencio, M., Carlomagno, Y., et al. (2019). Heterochromatin anomalies and double-stranded RNA accumulation underlie *C9orf72* poly(PR) toxicity. Science *363*, 6428.

STAR★METHODS

KEY RESOURCES TABLE

REAGENT or RESOURCE	SOURCE	IDENTIFIER
Antibodies		
Mouse monoclonal anti-HP1α	Euromedex	2HP-1H5-AS
Rat monoclonal anti-RNAPII Ser5p	ActiveMotif	61085; RRID:AB_2687451
Rabbit polyclonal anti-H3K9me3	Abcam	ab8898; RRID:AB_306848
Rabbit polyclonal anti-H3K27ac	Abcam	ab4729; RRID:AB_2118291
Rabbit polyclonal anti-GFP/CFP	Abcam	ab290; RRID:AB_303395
Rabbit polyclonal anti-TagRFP/TagBFP	Evrogen	AB233; RRID:AB_2571743
Biological Samples		
Suv39h double-null iMEFs	Peters et al., 2001	
NIH 3T3 GFP-HP1α	Müller et al., 2009	
U2OS 2-6-3 (with *lac*O/*tet*O reporter array)	Janicki et al., 2004	
Plasmids		
pGFP-HP1α/β/γ	Müller-Ott et al., 2014	
pMECP2-GFP	Müller-Ott et al., 2014	
pMBD1-GFP	Müller-Ott et al., 2014	
pGFP-NCL	Caudron-Herger et al., 2015	
pGFP-NPM	Caudron-Herger et al., 2015	
pTetR-RFP	Rademacher et al., 2017	
pLacI-GBP	Rothbauer et al., 2008	
pLacI-GFP	Jegou et al., 2009	
pGFP-PML III	Jegou et al., 2009	
pCMV-Tet3G	Clontech	
Critical Commercial Assays		
Thrombin cleavage kit	Millipore	69022-3
Factor Xa cleavage kit	Millipore	69037-3
Salmon Sperm DNA, low molecular weight	Sigma	31149, Lot # BCBS9523V
Software and Algorithms		
R	R Core Team, 2017	
EBImage (R package)	Pau et al., 2010	
STCor	Müller-Ott et al., 2014	

LEAD CONTACT AND MATERIALS AVAILABILITY

The Lead Contact for this study is Fabian Erdel (fabian.erdel@ibcg.biotoul.fr). All unique/stable reagents generated in this study are available from the corresponding authors with a completed Material Transfer Agreement.

EXPERIMENTAL MODEL AND SUBJECT DETAILS

Cells were grown in GIBCO DMEM (Thermo Fisher Scientific) supplemented with 10% fetal calf serum (PAA), 2 mM L-glutamine, 1% penicillin/streptomycin (PAA) and 1 g/l glucose for U2OS or 4.5 g/l glucose for iMEF and 3T3 cells. Cells were cultured at 37°C and 5% CO_2. References to the descriptions and the sources of the cell lines are given in the Key Resources Table above. Cell lines were generated and initially characterized in the respective laboratories. We tested them for the absence of mycoplasma with the Venor-GeM Advance kit (Minerva Biolabs) and assessed their authenticity by analyzing RNA-seq data generated with them as compared to published datasets.

METHOD DETAILS

Plasmids

For purification of recombinant mouse HP1, the coding sequences for mouse HP1α and GFP-HP1α were cloned into pET16 (coding for an N-terminal His-tag followed by a factor Xa site) and pET28 (coding for an N-terminal His-tag followed by a thrombin site), respectively. The H2B-GFP plasmid was cloned based on the histone constructs described previously (Jegou et al., 2009). The constructs encoding GFP-HP1α, GFP-HP1β, GFP-HP1γ, MBD1-GFP and MECP2-GFP (Müller-Ott et al., 2014), LacI-GBP (Rothbauer et al., 2008), LacI-GFP and GFP-PML III (Jegou et al., 2009), TetR-RFP (Rademacher et al., 2017) as well as GFP-NCL and GFP-NPM (Caudron-Herger et al., 2015) for expression in mammalian cells have been described previously in the indicated references. PHR-mCherry fusions with different candidate proteins for optodroplet experiments were constructed by inserting the coding region of the respective candidate (listed above) into a PHR-mCherry plasmid (Kennedy et al., 2010) to yield PHR-mCherry-tagged HP1α, HP1β, HP1γ, NCL and NPM. PHR-mCherry-HP1α-I163A was constructed based on PHR-mCherry-HP1α using site-directed mutagenesis following standard protocols. The phosphomimetic nE-HP1α mutant was constructed from a gBlock gene fragment (IDT) encoding the serine to glutamic acid mutations in the N-terminal extension described previously (Larson et al., 2017). The nE-HP1α-ΔCTE mutant was constructed based on nE-HP1α by deleting the C terminus as described (Larson et al., 2017). PHR-mCherry-SENP7 PxVxL was constructed from a gBlock gene fragment (Integrated DNA Technologies) encompassing aa 80-180 according to a published sequence (Romeo et al., 2015). PHR-mCherry-FUSN-HP1α was constructed by inserting FUSN from Addgene plasmid #122148 upstream of the coding sequence of HP1. PHR-mCherry-RGG_2-HP1α was constructed by inserting RGG-GFP-RGG from Addgene plasmid #124939 upstream of the coding sequence of HP1. The plasmid encoding for HP1α and ZsGreen (separated by an IRES) was constructed by inserting the coding sequence of HP1α into the cloning site of pTRE3G-ZsGreen1 (Clontech). The resulting pTRE3G-ZsGreen1-HP1α plasmid was cotransfeced with pCMV-Tet3G (Clontech) to induce expression. For dCas9-GFP ("dCas9-mock"), the dCas9 open reading frame derived from Addgene plasmid #60910 was cloned into a pEGFP-N1 backbone (Clontech). For dCas9-GFP-VPR, the coding sequence for VPR from Addgene plasmid #63798 was inserted downstream of the coding sequence for dCas9-GFP. The plasmid encoding for different single guide RNAs (sgRNAs) was derived from Addgene #61424. Inserted sgRNA targeting regions were: 5-'GGGCAAGAAAACTGAAAATCA-3′ (mSat) and 5′-GTTCGCTCACAATTCCACATG-3′ (mock, targeting the bacterial lacO sequence).

Protein expression, *in vitro* droplet formation and Western Blotting

Mouse HP1α and GFP-HP1α carrying an N-terminal His-tag were expressed in *E. coli* Rosetta cells. Cells were grown in LB medium supplemented with 1 mM IPTG at 18°C overnight. Subsequently, cells were pelleted and resuspended in lysis buffer (150 mM NaH_2PO_4, 300 mM NaCl, 10 mM imidazole, 25% glycerol, 4% sarkosyl, 1000 U benzonase, 1 μg/ml lysozyme, 0.5% Triton X-100, 1 mM DTT, 0.1 mM PMSF). Cleared cell lysates were incubated with 5 mL Ni-NTA resin (Macherey-Nagel) per 400 mL of bacterial culture and were allowed to bind for 2h at 4°C. The resin was centrifuged, washed and eluted with elution buffer (20 mM HEPES pH 7.8, 150 mM KCl, 400 mM imidazole, 10% glycerol, 1 mM DTT). Eluates were dialyzed into storage buffer without glycerol (20 mM HEPES pH 7.8, 200 mM KCl, 1 mM DTT). For the images in Figure 1A, proteins were cleaved with thrombin (1 U/1 mg protein) or factor Xa (1 μg/50 μg protein) at 4°C overnight (thrombin and factor Xa cleavage kits were obtained from Millipore). Thrombin or factor Xa were removed from the reaction with streptavidin agarose or XArrest agarose, respectively. Finally, proteins were concentrated with spin columns, supplemented with 10% glycerol, snap-frozen and stored at −80°C. HP1 protein concentrations were determined either with the BCA (bicinchoninic acid) protein assay or from absorbance measurements at 280 nm, using extinction coefficients ε = 29,450 M^{-1} cm^{-1} for HP1α and ε = 76,634 M^{-1} cm^{-1} for GFP-HP1α (both methods yielded comparable values).

Droplet formation was evaluated by mixing HP1 solutions with a 33 mg/ml solution of salmon sperm DNA (Sigma) in storage buffer and measuring the turbidity at a wavelength of 600 nm with a microplate reader (Thermo Fisher Scientific). For the images in Figure 1A, droplets were imaged on a Zeiss Axio Observer widefield microscope, using 1-2 μl HP1 solution (~400 μM) mixed with the same volume of DNA solution on a chambered glass slide at room temperature. For the images in Figure 1C and Figure S1, droplets were visualized by confocal microscopy using an Andor Dragonfly system equipped with 10x and 40x objectives, or a Leica TCS SP8 system equipped with a 63x objective.

For Western Blotting in Figure S4, cells were lysed in RIPA buffer, lysates were subjected to gel electrophoresis with precast 4−20% polyacrylamide gels (Bio-Rad) and blotted onto a nitrocellulose membrane (via semi-dry blotting). After blocking in Tris-buffered saline (TBS) with 5% milk, proteins were detected with primary antibodies against GFP/CFP or TagRFP/TagBFP (both diluted in TBS with 1% BSA) and HRP-linked anti-rabbit IgG (in TBS with 5% milk). Bands were detected by chemoluminescence using clarity western ECL substrate (Bio-Rad).

Immunostaining and transfection

For immunostaining, cells were seeded onto coverslips (borosilicate #1.5, Thermo Fisher Scientific) and fixed with 4% paraformaldehyde for 12 minutes, permeabilized with 0.2% Triton X-100 in PBS for 12 minutes, and blocked with 5% BSA/0.1% Triton X-100 in PBS for 30 minutes (for STED) or 10% goat serum in PBS (for confocal microscopy). Cells were labeled with 1:100 diluted primary antibodies and with secondary antibodies conjugated with Abberior StarRed (for STED) or Alexa488/Alexa568 (for confocal microscopy) in blocking buffer for one hour. After 3 washes with PBS, DNA was stained with DAPI (1 μg/ml, 4 minutes, for STED)

and coverslips were rinsed with water (3 washes, 5 minutes each), dehydrated in 100% ethanol and mounted with Mowiol (for STED) or Prolong Diamond (for confocal microscopy).

iMEF cells were transfected with Lipofectamine 3000 (Thermo Fisher Scientific) according to the manufacturer's protocol, U2OS cells were transfected with Effectene (QIAGEN) or Xtreme Gene 9 (Roche) according to the manufacturers' protocols. For recruitment of TetR-PHR-YFP-HP1 to *tet*O sites (Figure 4E), cells were cultured in medium containing 500 ng/ml doxycycline (Sigma). More information about this experiment is provided in the qPCR section below. For the analysis of chromocenters in response to increased HP1α levels (Figures 7A–7C), iMEF cells were transfected with pTRE3G-ZsGreen1-HP1α and pCMV-Tet3G and were grown in medium supplemented with 500 ng/ml doxycycline for 24 hours. Subsequently, cells were fixed, stained and mounted as described above. For dCas9 recruitment to major satellite sequences (Figures 7D–7I), cells were transfected with dCas9-VPR or dCas9-mock and a plasmid encoding for a guide RNA targeting major satellite repeats (see sequence above). After 30 hours, cells were fixed, stained and mounted.

Confocal and STED microscopy of living and fixed cells

Confocal imaging experiments in Figures 3, 4, 5, and 6 were conducted using a Leica TCS SP5 II confocal microscope equipped with a 63x oil immersion objective. The images of cells expressing dCas9-constructs (Figure 7) were acquired using an Andor Dragonfly 505 spinning disk microscope with a 100x silicone immersion objective. The STED microscope used for the images in Figure 2 is similar to the one published in Gorlitz et al., with a 594 nm and a 650 nm excitation laser combined with a 40 MHz pulsed 775 nm STED laser (Gorlitz et al., 2014). A pinhole size of 80 μm and a pixel size of 15 nm was used.

Optodroplet induction and stability measurements

Optodroplets in Figure 3E and Figure S3B were induced by scanning 512 × 512 pixel images with a 488 nm laser for 25 s, and PHR-mCherry fusions were imaged using a 561 nm laser. Laser intensities were kept constant for all constructs. To ensure comparability across different days of experimentation, HP1α and HP1α I163A were measured on each day as controls. To measure optodroplet lifetimes in Figure 3F and Figure S3C, cells with rather high expression levels were used to ensure robust optodroplet formation. Compared to the settings for optodroplet induction, cells were illuminated with a 488 nm laser at ~5-fold higher intensity for an additional 25 s to induce large numbers of optodroplets. Subsequently, cells were imaged with a 561 nm laser (time resolution: 10 s per frame).

Protein mobility measurements

Fluorescence recovery after photobleaching (FRAP) experiments were conducted using a Leica TCS SP5 II confocal microscope equipped with a 63x oil immersion objective. All half-chromocenter and half-nucleolus FRAP experiments were conducted with a line frequency of 400 Hz, an image size of 512 × 64 pixels, a pixel size of 60 nm, no line/frame averaging, and one bleach frame with all Argon laser lines at full laser power (~2 mW in the back focal plane of the objective). The left half of chromocenters/nucleoli was bleached in all experiments to avoid potential differences due to an altered scan direction.

Conventional fluorescence correlation spectroscopy (FCS) experiments to determine protein concentrations in optodroplet assays (Figure 3D) were conducted as previously described (Müller-Ott et al., 2014). A Zeiss LSM 710 ConfoCor 3 microscope with a 40x water immersion objective was used, and expression levels were determined based on the amplitudes of the correlation functions.

Polarization-sensitive fluorescence correlation spectroscopy (Pol-FCS) experiments (Figures 6D–6H) were conducted using a previously described setup (Grußmayer and Herten, 2017) with an additional polarizer in the excitation beam path and a polarizing beam splitter behind the pinhole, yielding a similar configuration as described in (Oura et al., 2016). Measurements were conducted for 2-5 minutes in 3T3 cells stably expressing GFP-HP1 (Müller et al., 2009). Immobile GFP-HP1 molecules were bleached beforehand by illuminating the selected position for 10 s. Correlation curves were calculated as described in the section on quantification below.

Measurement of reporter activity by real-time quantitative PCR

To test the repressive potential of HP1 at the reporter array (Figure 4E), cells were transfected with BFP-LacI-VP16 to activate the reporter. After 24 hours, doxycycline was added to the medium (500 ng/ml) to tether HP1 to the array, and for optodroplet induction cells were cultured in the presence of light. After 72 hours, RNA ($\geq$200 nt) was isolated with the NucleoSpin RNA Plus kit (Macherey-Nagel) and digested with DNase I (Promega) for 30 min at 37°C. The cDNA was synthesized using Superscript IV reverse transcriptase (Thermo Fisher Scientific) and RNA was digested using RNase H. The qPCR reaction was performed using the SYBR Green mix (Applied Biosystems) and primers specific for the CFP-SKL reporter (fwd: 5′-GTC CGG ACT CAG ATC TCG A-3′ and rev: 5′-TTC AAA GCT TGG ACT GCA GG-3′) with the following program: 10 min, 95°C, and 40 cycles of 1 min, 60°C. RNA was quantified using a standard dilution and normalized to average "pre" levels before illumination.

QUANTIFICATION AND STATISTICAL ANALYSIS

Turbidity analysis

Turbidity values of HP1-DNA mixtures in Figure 1B were normalized by subtracting the turbidity of the buffer. The Hill function used for fitting the normalized turbidity $t(c)$ reads

$$t(c) = \frac{a}{1 + \left(\frac{c_{sat}}{c}\right)^n}$$

Here, a is the turbidity for infinite HP1 concentration (upper plateau), c_{sat} is the apparent half-saturation concentration, and n is the Hill coefficient.

Image correlation spectroscopy

Image correlation spectroscopy analysis was conducted in R (R Core Team, 2017) using the EBImage package (Pau et al., 2010). The method uses intensity fluctuations in an image to quantitate the characteristic size of structures that are present in the image (Petersen et al., 1993). To this end, a correlation function was calculated based on the pixel intensities, whose decay lengths correspond to the sizes of the structures of interest. The first step in the analysis was to segment nuclei using a 40% quantile threshold, and subsequently chromocenters were segmented using a 90% quantile threshold with respect to the pixel intensities in the nucleus. The segmented areas were made rectangular by filling surrounding pixels with the average intensity in the segmented area, ensuring that no intensity fluctuations arise from these pixels. The resulting rectangular images were then shifted with respect to themselves in x- and y-direction to calculate the following correlation function

$$G(\Delta x, \Delta y) = \frac{\langle (I(x,y) - \langle I \rangle) \cdot (I(x+\Delta x, y+\Delta y) - \langle I \rangle) \rangle}{\langle I \rangle^2}$$

Here, $\langle \ldots \rangle$ denotes averaging, $I(x,y)$ is the intensity at pixel (x,y), $\langle I \rangle$ is the average intensity of the entire image, and Δx and Δy are the spatial shifts. The two-dimensional correlation function $G(\Delta x, \Delta y)$ was transformed into a one-dimensional correlation function $G(d)$ by averaging all $G(\Delta x, \Delta y)$ values with the same shift length $d = \sqrt{\Delta x^2 + \Delta y^2}$, regardless of shift direction. The resulting one-dimensional correlation functions were fitted with the following equation

$$G(d) = a_1 \cdot \exp\left(-\left(\frac{d}{\lambda_1}\right)^{n_1}\right) + a_2 \cdot \exp\left(-\left(\frac{d}{\lambda_2}\right)^{n_2}\right)$$

Here, a_1 and a_2 are the relative contributions from the two components, λ_1 and λ_2 represent the correlation lengths that correspond to the radii of the objects, and n_1 and n_2 describe the "fuzziness" of each component. Large values for n reflect equally sized objects with sharp boundaries and small values for n reflect broader distributions of object sizes and/or less distinct boundaries. Curves for wild-type cells had to be fitted with two components while curves for *Suv39h* dn cells could be fitted well without the second component (i.e., with $a_2 = 0$). The values reported in Figure 2D correspond to the full diameters of the small structures ($2 \cdot \lambda_1$). Fit parameters are listed in Table S2.

Quantification of optodroplet abundance and lifetime

The abundance of optodroplets in Figure 3 was quantified by calculating the coefficient of variation (CV, standard deviation over mean) for the pixel intensities in the nucleoplasm. Segmentation and quantification was done in R (R Core Team, 2017) using the EBImage package (Pau et al., 2010). For the analysis in Figure 3E, the CV of the image before illumination was subtracted from the CV of the image after illumination to obtain the relative droplet abundance. For the time series in Figure 3F, CV values were calculated for each image, divided by the CV of the first image, and finally the CV of the last image was subtracted, yielding "double-normalized" curves. This procedure corrects for preexisting heterogeneous structures that are unrelated to optodroplets. If the CV before illumination was lower than the CV in the last image, the CV before illumination was subtracted instead to account for residual droplets that have not dissociated during the experiment. For nucleolar proteins (NCL and NPM), pixels residing in intensely labeled nucleoli were removed prior to the analysis. The same was done for large aggregates that were occasionally observed before optodroplet induction for FUSN-HP1, RGG_2-HP1 and the phosphomimetic HP1 variants. For RGG_2-HP1, cells with very low expression levels were used for the analysis, as cells with intermediate and high expression levels tended to contain many aggregates. To remove the contribution of noise to the CV of the respective RGG_2-HP1 images, the latter were smoothed before the analysis (using a Gaussian with sigma = 2, gblur-function in EBImage). The resulting curves were fitted with the following equation, which describes a two-step dissociation process that produces the short lag phase observed in the decay curves for early time points:

$$A(t) = A_0 \cdot (1 + kt) \cdot \exp(-kt)$$

Here, A is the abundance of optodroplets, A_0 is the initial abundance, and k is the decay rate. The lifetime τ reported in Figure 3 corresponds to $\tau = 1.68/k$, which is the time at which the abundance has dropped to $A_0/2$. An additional plateau was added to the equation for proteins forming long-lived droplets (FUSN-HP1 and RGG_2-HP1).

FRAP analysis of HP1 at lacO-array

The FRAP curves in Figure 4D and Figure S4A were acquired at a time resolution of 2 s per frame. We fitted the curve for HP1 with two exponential functions and neglected diffusion. The time constant for the second component was fixed to the result obtained

for the GFP control (Figure S4) to increase the robustness of the fit. With this approach, the size of the transient and stable protein fractions could reliably be measured without resolving diffusion and binding contributions for the transient fraction.

FRAP analysis of half-bleached cellular structures

To fit the FRAP data in Figure 5, image series were registered using a custom-written R script. Subsequently, FRAP curves for the bleached region of interest (ROI) and the non-bleached ROI of the structure of interest were calculated according to

$$\mathrm{FRAP}_{\mathrm{B/NB}}(t) = \frac{\langle I_{\mathrm{B/NB}}\rangle - \langle I_{\mathrm{BG}}\rangle}{\langle I_{\mathrm{Nucleus}}\rangle - \langle I_{\mathrm{BG}}\rangle}$$

Here, $\langle I_{\mathrm{B}}\rangle$, $\langle I_{\mathrm{NB}}\rangle$, $\langle I_{\mathrm{Nucleus}}\rangle$ and $\langle I_{\mathrm{BG}}\rangle$ denote the average intensities in the bleached ROI, the non-bleached ROI, the background of the image, and the imaged part of the nucleoplasm, respectively. Next, the FRAP signals for the bleached and non-bleached ROIs were multiplied with the relative size of the respective ROI to obtain quantities that are proportional to the number of bleached/non-bleached particles in the respective ROI:

$$\mathrm{FRAP}'_{\mathrm{B/NB}}(t) = \frac{N_{\mathrm{B/NB}}}{N_{\mathrm{B}} + N_{\mathrm{NB}}}\mathrm{FRAP}_{\mathrm{B/NB}}(t)$$

Here, N_{B} and N_{NB} are the number of pixels of the bleached and non-bleached ROI, respectively. Next, both signals were normalized with respect to the number of bleached molecules:

$$\mathrm{FRAP}''_{\mathrm{B/NB}}(t) = \frac{\mathrm{FRAP}'_{\mathrm{B/NB}}(t) - \mathrm{FRAP}'_{\mathrm{B}}(t_{\mathrm{bleach}})}{\langle \mathrm{FRAP}'_{\mathrm{B}}(t_{\mathrm{pre}})\rangle - \mathrm{FRAP}'_{\mathrm{B}}(t_{\mathrm{bleach}})}$$

Here, t_{bleach} is the bleach frame and $\langle \mathrm{FRAP}'_{\mathrm{B}}(t_{\mathrm{pre}})\rangle$ is the average signal in the bleached ROI before the bleach takes place. Thus, the FRAP curve for the bleached ROI was double-normalized, i.e., it equals unity before the bleach and zero in the first post-bleach frame. Note that both the signal for the bleached and for the non-bleached ROI were scaled equally (with respect to the bleached ROI) to preserve their relative magnitude that scales with the number of particles in each ROI. Finally, an additive offset was applied to the signal in the non-bleached ROI to normalize it to unity before the bleach

$$\mathrm{FRAP}'''_{\mathrm{NB}}(t) = \mathrm{FRAP}''_{\mathrm{NB}}(t) - \langle \mathrm{FRAP}''_{\mathrm{NB}}(t_{\mathrm{pre}})\rangle + 1$$

The resulting signals, $\mathrm{FRAP}''_{\mathrm{B}}(t)$ and $\mathrm{FRAP}'''_{\mathrm{NB}}(t)$, are proportional to the net change of the number of fluorescent particles in the respective ROIs.

To fit the normalized curves, a confined diffusion model was used, which assumes (effective) unobstructed diffusion within the structure of interest and a barrier at the boundary of the structure. Effective diffusion implicitly takes into account potential contributions from transient binding interactions that occur within the structure (Müller-Ott et al., 2014). The two main fit parameters are the barrier height h and the (effective) diffusion time τ_{D}. The inverse of the barrier height h can be interpreted as the effective thickness l of the boundary, i.e., diffusion across the boundary is equivalent to unobstructed diffusion over the distance l. In Figure 5, the dimensionless ratio $p = R/l$ between radius R and effective boundary thickness l is reported. It resembles a permeability with $p = 0$ for an impermeable boundary and $p = \infty$ for the absence of any boundary.

For simplicity, the recovery process was treated in two dimensions. The following equation solves the two-dimensional diffusion problem with a barrier of height h at the boundary of a circular domain with radius R (Carslaw and Jaeger, 1959):

$$\mathrm{c}(r,\varphi,t) = \frac{1}{\pi R^2}\sum_{n=-\infty}^{\infty}\sum_{\alpha_n}\frac{\alpha_n^2\ J_n(\alpha_n r)e^{-\alpha_n^2\ t/\tau_{\mathrm{D}}}}{\left(\alpha_n^2 + h^2 - \frac{n^2}{R^2}\right)J_n^2(\alpha_n R)}\int_0^{2\pi} d\varphi'\ \cos(n(\varphi-\varphi'))\int_0^R r'\,dr'\,c_0(r',\varphi')\,J_n(\alpha_n r')$$

Here, $\mathrm{c}(r,\varphi,t)$ is the concentration of bleached particles over time, $c_0(r',\varphi')$ is the initial distribution of bleached particles, J_n is the Bessel function of the first kind of order n, and α_n are the positive roots of $J'_n(\alpha_n R) + hJ_n(\alpha_n R) = 0$. With the initial distribution $c_0(r',\varphi') = \frac{2\Theta(\varphi'-\pi)}{\pi R^2}$ (where Θ denotes the Heaviside step function), which corresponds to one half of the circle being initially bleached, the following expression is obtained

$$\mathrm{c}(r,\varphi,t) = \frac{4}{\pi^2 R^2}\sum_{n=-\infty}^{\infty}\sum_{\alpha_n}\frac{\alpha_n^2\left(\frac{\alpha_n R}{2}\right)^n\cos\left(\frac{3n\pi}{2} - n\varphi\right)\sin\left(\frac{n\pi}{2}\right)J_n(\alpha_n r)\mathrm{PFQ}_n(\alpha_n R)\,e^{-\alpha_n^2\ t/\tau_{\mathrm{D}}}}{\left(\alpha_n^2 + h^2 - \frac{n^2}{R^2}\right)n^2(2+n)\ \Gamma(n)J_n^2(\alpha_n R)}$$

Here, the hypergeometric function $\mathrm{PFQ}_n(\alpha_n R) = {}_1\mathrm{F}_2\left(1+\frac{n}{2}; 1+n, 2+\frac{n}{2}; -\frac{\alpha_n^2 R^2}{4}\right)$ is used. For $\varphi = \pm(\pi/2)$, this equation yields the time evolution of the spatial intensity profile perpendicular to the line that separates the bleached from the non-bleached half of the circle (it can be multiplied with $(\pi R^2/2)$ to obtain normalized profiles). For the integrated intensity in both semicircles, the following expressions are obtained

$$c_{\rm B}(t) = \int_{\pi}^{2\pi} d\varphi \int_0^R r\,dr\,c(r,\varphi,t) = \frac{8}{\pi^2}\sum_{n=-\infty}^{\infty}\sum_{\alpha_n}\frac{\alpha_n^2\left(\frac{\alpha_n R}{2}\right)^{2n}\sin^2\left(\frac{n\pi}{2}\right){\rm PFQ}_n^2(\alpha_n R)\,e^{-\alpha_n^2\,t/\tau_{\rm D}}}{\left(\alpha_n^2+h^2-\frac{n^2}{R^2}\right)n^4(2+n)^2\,\Gamma(n)^2\,J_n^2(\alpha_n R)}$$

$$c_{\rm NB}(t) = \int_0^{\pi} d\varphi \int_0^R r\,dr\,c(r,\varphi,t) = \frac{8}{\pi^2}\sum_{n=-\infty}^{\infty}\sum_{\alpha_n}\frac{\alpha_n^2\left(\frac{\alpha_n R}{2}\right)^{2n}\sin^2\left(\frac{n\pi}{2}\right)\cos(n\pi){\rm PFQ}_n^2(\alpha_n R)\,e^{-\alpha_n^2\,t/\tau_{\rm D}}}{\left(\alpha_n^2+h^2-\frac{n^2}{R^2}\right)n^4(2+n)^2\,\Gamma(n)^2\,J_n^2(\alpha_n R)}$$

Note that for $n \neq 0$ the summands with even n vanish, whereas summands with odd n have the same absolute value but a different sign for $c_{\rm B}$ and $c_{\rm NB}$ (due to the additional $\cos(n\pi)$ term). These terms describe the particle exchange between the bleached and the non-bleached half of the circle. For $n = 0$, the summands for both $c_{\rm B}$ and $c_{\rm NB}$ equal

$$c_{\rm B/NB}^{n=0}(t) = \frac{2}{R^2}\sum_{\alpha_0}\frac{h^2\,e^{-\alpha_0^2\,t/\tau_{\rm D}}}{\alpha_0^2\left(\alpha_0^2+h^2\right)}$$

This term describes the particle exchange across the boundary of the circle. For fitting the experimental curves in Figure 5, the resulting equations were multiplied with a scaling factor, and an additive immobile fraction was taken into account.

Pol-FCS analysis

For Pol-FCS, cross-correlation functions of detectors measuring the same polarization contain contributions from rotational and translational diffusion, whereas cross-correlation functions of detectors measuring crossed polarizations lack the rotational contribution. The curves in Figure 6D represent averages of cross-correlation functions of all detector pairs measuring crossed polarizations (4 pairs). The curves in Figures 6E and 6G represent cross-correlation functions of detectors measuring the polarization parallel to the excitation laser beam. Slow intensity fluctuations and vibrations (> 100 ms) were removed from the signal, and quasi-logarithmic correlation functions were calculated for windows of 5 s with custom-written Java code that was derived from STCor (Müller-Ott et al., 2014). The Pol-FCS curves in Figures 6D and 6E were globally fitted with the following functions

$$G_{\rm parallel}(\tau) = G_{\rm rot}(\tau)G_{\rm trip}(\tau)G_{\rm trans}(\tau)$$

$$G_{\rm crossed}(\tau) = G_{\rm trip}(\tau)G_{\rm trans}(\tau)$$

The individual contributions for rotational diffusion $G_{\rm rot}(\tau)$, triplet $G_{\rm trip}(\tau)$ and translational diffusion $G_{\rm trans}(\tau)$ read

$$G_{\rm rot}(\tau) = \left(1 + f_{\rm R}\exp\left(-\frac{\tau}{\tau_{\rm R}}\right)\right)$$

$$G_{\rm trip}(\tau) = \left(1 + f_{\rm T}\exp\left(-\frac{\tau}{\tau_{\rm T}}\right)\right)$$

$$G_{\rm trans}(\tau) = \frac{f_1}{N}\left(1+\left(\frac{\tau}{\tau_{\rm D1}}\right)^{\alpha_1}\right)^{-1}\left(1+\frac{1}{\kappa^2}\left(\frac{\tau}{\tau_{\rm D1}}\right)^{\alpha_1}\right)^{-\frac{1}{2}}+\frac{1-f_1}{N}\left(1+\left(\frac{\tau}{\tau_{\rm D2}}\right)^{\alpha_2}\right)^{-1}\left(1+\frac{1}{\kappa^2}\left(\frac{\tau}{\tau_{\rm D2}}\right)^{\alpha_2}\right)^{-\frac{1}{2}}$$

Here, N is the particle number, $\tau_{\rm R}$, $\tau_{\rm T}$, $\tau_{\rm D1}$ and $\tau_{\rm D2}$ are the rotational correlation time, triplet time, translational diffusion time for the fast and for the slow component, respectively, $f_{\rm R}$, $f_{\rm T}$ and f_1 are the contributions from rotation, triplet and fast translational diffusion, α_1 and α_2 are the anomaly parameters for fast and slow translational diffusion, respectively, and κ is a structural parameter characterizing the geometry of the microscope's focus that we fixed for the fit. The Pol-FCS curves for GFP-HP1 in the cytoplasm in Figures 6D and 6E, and the curves for GFP-HP1 in glycerol-water mixtures in Figure 6G could be fitted with only one translational diffusion component (i.e., f_1 = 1). Correlation curves for translational diffusion of GFP-HP1 have been fitted previously with the translational part of this function, with the slow component representing motion of HP1-bound chromatin (Müller-Ott et al., 2014). The additional rotational term could be robustly determined (i) because the rotational correlation time is much smaller than the translational diffusion time and thus well separated from the translational part, and (ii) because the rotational term is the only term that distinguishes the curves in Figure 6E from those in Figure 6D. Thus, the triplet contribution and the translational contribution in the global fit were mostly determined by the curves in Figure 6D and the rotational contribution by the additional decay in the curves in Figure 6E.

Intensity analysis of confocal images for dose-response relationships

The dose-response relationships for HP1 overexpression and dCas9-VPR recruitment in Figure 7 were quantified with R (R Core Team, 2017) using the EBImage package (Pau et al., 2010). For the analysis in Figure 7C, we segmented nuclei in the HP1 channel based on the threshold obtained by Otsu's method (Otsu, 1979) and then segmented chromocenters in the DAPI channel using a threshold of 1.3-times the median DAPI intensity in each nucleus. The resulting regions were used to measure the chromocenter

size and the average intensities in the different channels. To resolve the dose-response relationship, we divided the ZsGreen intensities into three groups (Figure S7A). The cutoffs were chosen to yield a good compromise between similarly sized groups and similarly spaced ZsGreen levels. For the analysis in Figures 7F and 7I, major satellite repeats were segmented based on the dCas9 channel using the following threshold for each nucleus: median + 0.1·(maximum-median). Subsequently, the size of major satellite repeats and the average intensities in the different channels were measured in the respective regions. To resolve dose-response relationships in Figures 7F and 7I as well as Figure S7C, we divided cells into three groups based on their dCas9 intensities (Figure S7B), with the cutoffs corresponding to the 40th and 60th percentile, and analyzed the DAPI and H3K27ac signals in each group of cells.

DATA AND CODE AVAILABILITY

This study did not generate datasets or code.

Molecular Cell, Volume *78*

Supplemental Information

Mouse Heterochromatin Adopts Digital Compaction States without Showing Hallmarks of HP1-Driven Liquid-Liquid Phase Separation

Fabian Erdel, Anne Rademacher, Rifka Vlijm, Jana Tünnermann, Lukas Frank, Robin Weinmann, Elisabeth Schweigert, Klaus Yserentant, Johan Hummert, Caroline Bauer, Sabrina Schumacher, Ahmad Al Alwash, Christophe Normand, Dirk-Peter Herten, Johann Engelhardt, and Karsten Rippe

Supplemental Figures

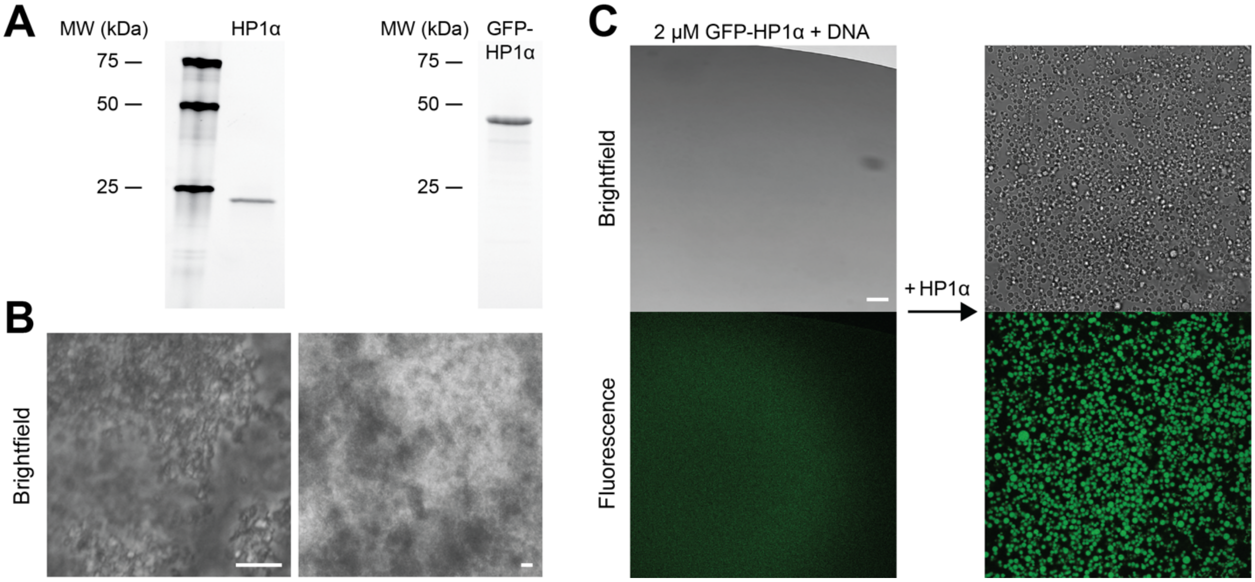

Figure S1. Characterization of recombinant mouse HP1α. Related to Fig. 1.

(**A**) SDS polyacrylamide gel electrophoresis of purified mouse HP1α and GFP-HP1α.
(**B**) Irregular structures in mixtures of HP1α and DNA that formed in addition to droplets, especially at very high HP1α concentrations. Scale bars, 10 μm.
(**C**) Left: No droplets formed in a mixture of 2 μM GFP-HP1α and saturating amounts of DNA. Right: Upon addition of a 180 μM solution of untagged HP1α to the same mixture under the microscope, green fluorescent droplets formed. The final concentrations after HP1α addition were 45 μM untagged HP1α and 1.5 μM GFP-HP1α. All components were dissolved in storage buffer (STAR Methods). Scale bar, 10 μm.

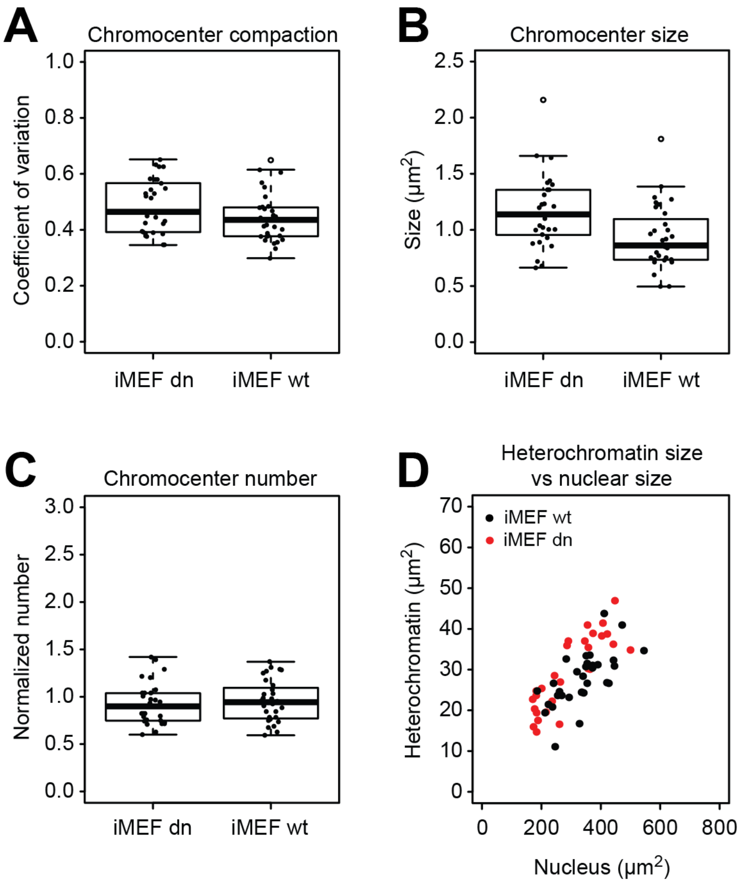

Figure S2. Compaction, size and number of chromocenters. Related to Fig. 2.

(**A**) Compaction of chromocenters assessed by the coefficient of variation of DAPI signals. A similar approach has previously been used to measure heterochromatin compaction (Casas-Delucchi et al., 2012).
(**B**) Size of chromocenters determined from DAPI signals.
(**C**) Number of chromocenters per 10 μm^2 nuclear area. As the number of chromocenters was positively correlated with the size of cell nuclei, the normalization was used to make the results independent of nuclear size.
(**D**) The area occupied by heterochromatin scales with the size of the nucleus. This is in contrast to the inverse scaling that has been reported for nucleoli in early *C. elegans* embryos (Weber and Brangwynne, 2015), which is expected for organelles formed by LLPS (assuming that the number of molecules that drive LLPS is the same in each nucleus).

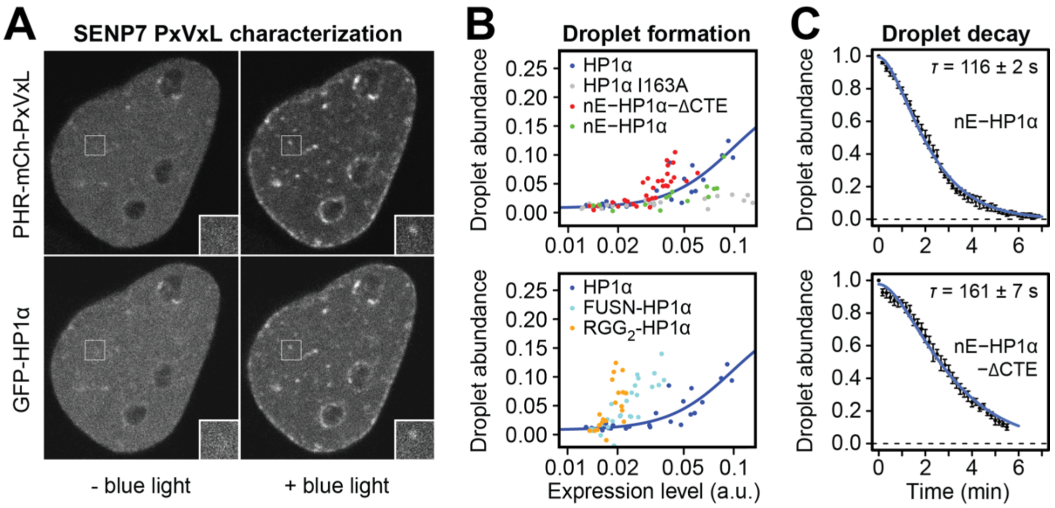

Figure S3. Properties of optodroplets containing different proteins. Related to Fig. 3.

(**A**) Confocal microscopy images of a representative U2OS cell that shows colocalization of GFP-HP1α and the PHR-mCherry-tagged PxVxL module of SENP7. Both proteins were located at perinucleolar regions before illumination with blue light (left) and assembled into optodroplets after illumination (right, see inset for a magnified optodroplet). Based on the GFP-HP1α signal in both images, heterochromatin regions already present before blue light exposure can be distinguished from light-induced optodroplets.
(**B**) Capacity of droplet formation for fusions of PHR-mCherry with the indicated proteins.
(**C**) Dissociation kinetics of optodroplets containing fusions of PHR-mCherry with the phosphomimetic HP1 variants nE-HP1α (top) and nE-HP1α-ΔCTE (bottom). Error bars represent s.e.m. from at least 10 replicates. Errors for half-lives represent standard fit errors.

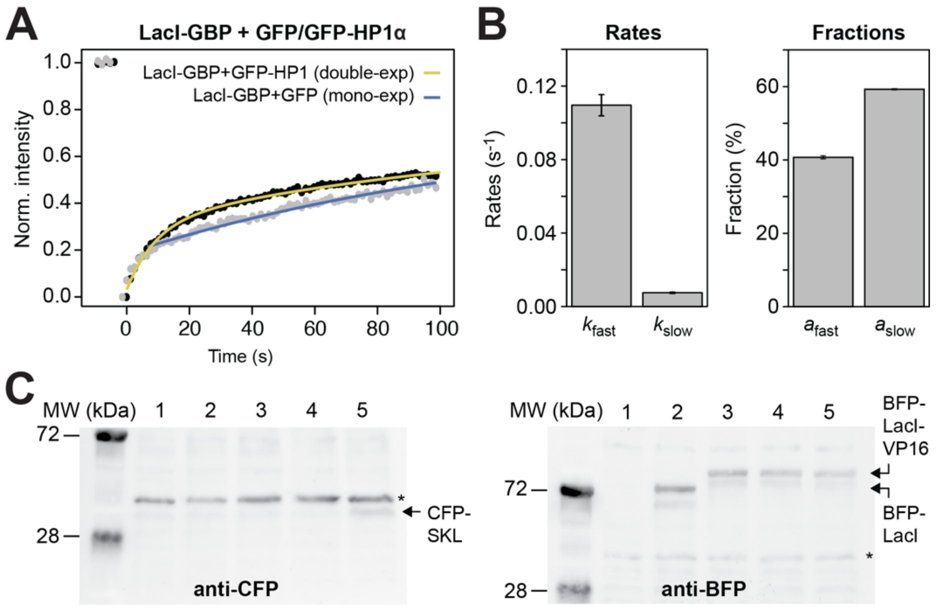

Figure S4. Protein exchange and activity at the reporter array. Related to Fig. 4.

(**A**) GFP bound to *lac*O arrays via LacI-GBP (GFP binding protein) was bleached to measure the exchange kinetics of directly recruited GFP-proteins. The data for GFP (gray points) and GFP-HP1α (black points) were globally fitted with a mono-exponential function for GFP (blue curve) and a double-exponential function for GFP-HP1α (yellow curve), with the rate for GFP being kept identical to the slow rate for GFP-HP1α. The early time points for GFP were not considered for the fit because they contain a contribution from diffusion of LacI-GBP•GFP, while the contribution from slow exchange of LacI-GBP•GFP at the *lac*O array is most prominent for later time points.

(**B**) Parameters obtained from the global fit in the previous panel. Error bars represent standard fit errors. k_{fast} is the rate for the fast component that is specific to GFP-HP1α; k_{slow} is the rate for the slower component that is present in both recovery curves; a_{fast} and a_{slow} are the sizes of the fast and the slow fraction for GFP-HP1α.

(**C**) Western Blot analysis of U2OS reporter cells under different conditions. The CFP-SKL protein visible in the left blot (probed with an antibody against GFP/CFP) is encoded by the reporter adjacent to the *lac*O/*tet*O sites (see (Janicki et al., 2004) for a detailed description of the construct). The right blot was probed with an antibody against BFP and shows the different transiently expressed BFP-fusions. The lanes correspond to the following conditions: 1: untransfected cells; 2: BFP-LacI-transfected cells; 3-5: BFP-LacI-VP16-transfected cells, with doxycycline and with light (3), with doxycycline and without light (4) and without doxycycline and without light (5). Asterisks indicate nonspecific bands.

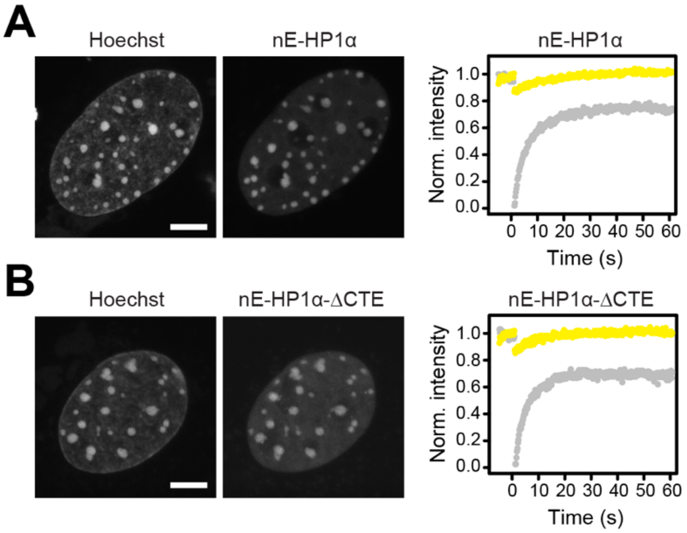

Figure S5. Internal mixing of phosphomimetic HP1α variants in chromocenters. Related to Fig. 5.

Localization and half-chromocenter bleach for the phosphomimetic HP1 variants GFP-nE-HP1α (panel **A**; *n* = 11 cells for the bleach experiment) and GFP-nE-HP1α-ΔCTE (panel **B**; *n* = 16 cells for the bleach experiment). No anti-correlation between the bleached and the non-bleached half was observed. Accordingly, the phosphomimetic HP1α variants behaved similarly to GFP-HP1α and displayed no preferential internal mixing in chromocenters. Bleach experiments were conducted in cells without Hoechst. Scale bars, 5 µm.

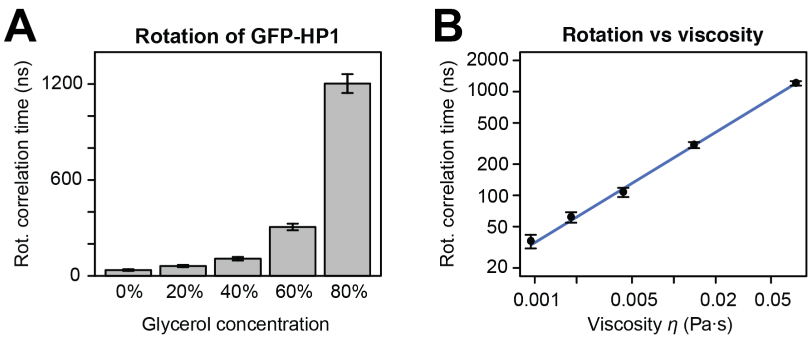

Figure S6. Relationship between viscosity and GFP-HP1 rotational correlation times *in vitro*. Related to Fig. 6.

Pol-FCS experiments were conducted with purified GFP-HP1 at a concentration of 50 nM in glycerol-water mixtures with the indicated glycerol concentrations.
(**A**) Rotational correlation times for GFP-HP1 in glycerol-water mixtures. Error bars represent standard fit errors.
(**B**) Relationship between GFP-HP1 rotational correlation times from the previous panel and the viscosity of the respective glycerol-water mixture taken from the literature (Cheng, 2008). The blue line is a fit with the equation $\tau_R/\tau_{R,0} = (\eta/\eta_0)^q$ (Lavalette et al., 1999), yielding $q = 0.82 \pm 0.01$ (the error is the standard fit error).

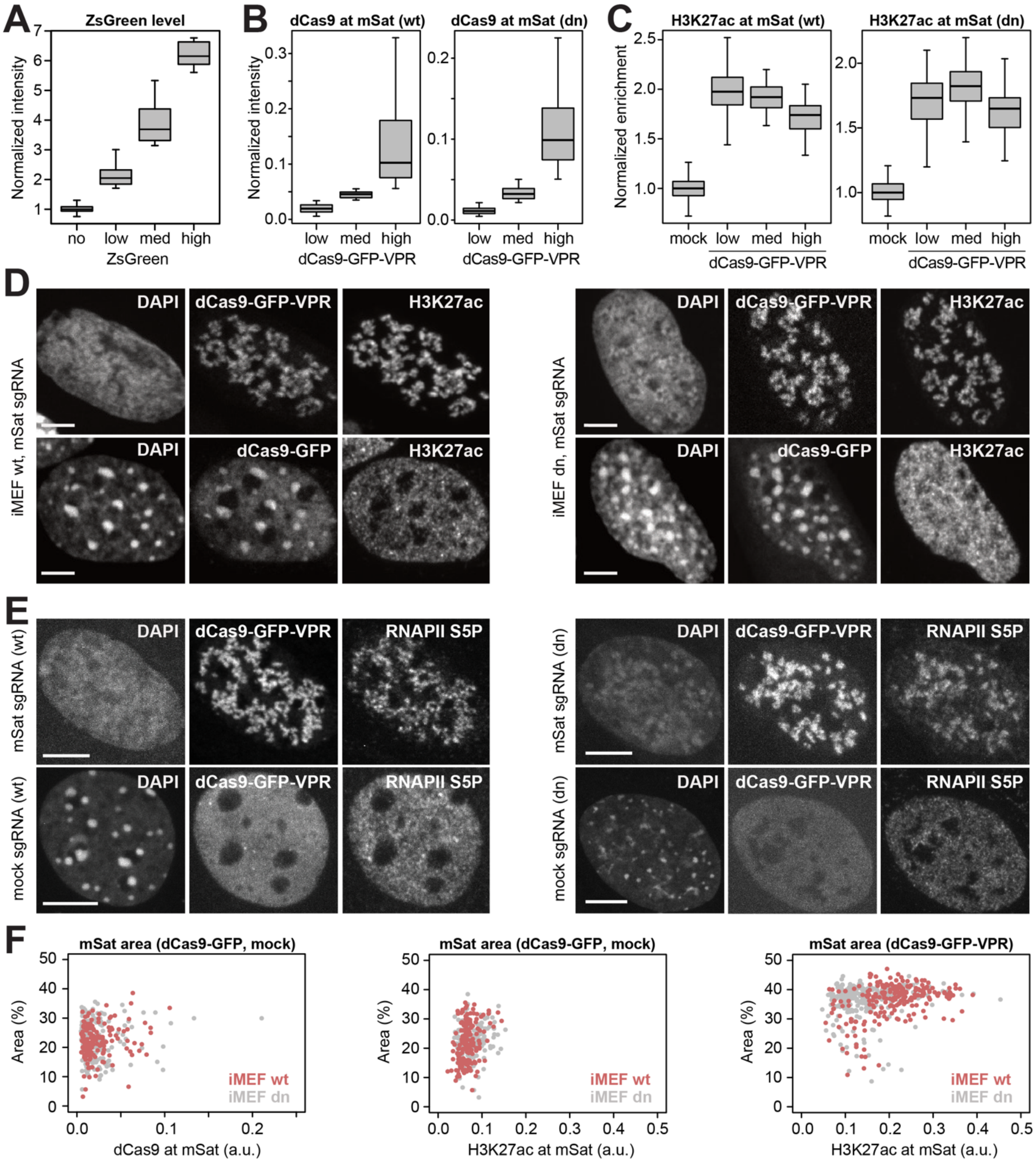

Figure S7. Properties of iMEF cells exposed to different perturbations. Related to Fig. 7.

(**A**) Expression level of ZsGreen for the individual groups of cells analyzed in **Fig. 7C**.
(**B**) Amount of dCas9 bound at major satellites for the individual groups of cells analyzed in **Fig. 7F** and **7I**.
(**C**) Amount of H3K27ac at major satellites for the individual groups of cells analyzed in **Fig. 7F** and **7I**.
(**D**) DAPI, dCas9 and H3K27ac signals for the cells shown in **Fig. 7E** (left) and for iMEF dn cells expressing the same constructs for comparison (right). Scale bars, 5 µm.
(**E**) DAPI, dCas9-GFP-VPR and RNA polymerase II phosphorylated at serine 5 (RNAPII S5P) signals for the cell shown in **Fig. 7H** (top left), for an iMEF wt cell expressing a mock sgRNA instead of an mSat sgRNA (bottom left), and for iMEF dn cells expressing the same constructs for comparison (right). Scale bars, 5 µm.
(**F**) Relationship between chromocenter size and dCas9-GFP (left) or H3K27ac (center/right) at major satellites in iMEF wt (red) and iMEF dn (gray) cells expressing dCas9-GFP (left/center) or dCas9-GFP-VPR (right).

Supplemental Tables

Fit parameter	HP1α	GFP-HP1α
c_{sat} (μM)	45 ± 4	23 ± 2
n	1.7 ± 0.1	1.5 ± 0.1
a	1.5 ± 0.1	

Table S1. Fit results for turbidity curves. Related to Fig. 1.

Parameters for Hill equation fits (STAR Methods) to the turbidity curves for HP1α and GFP-HP1α in **Fig. 1B**. Errors represent standard fit errors. The upper plateau (*a*) was globally fitted for both proteins, increasing the robustness of the fit.

Fit parameter	HP1α, iMEF wt	H3K9me3, iMEF wt	HP1α, iMEF dn	H3K9me3, iMEF dn
a_1	0.60 ± 0.05	0.74 ± 0.01	1	1
a_2	0.40 ± 0.05	0.26 ± 0.01	-	-
λ_1 (nm)	51 ± 9	60 ± 1	73 ± 2	75 ± 1
λ_2 (nm)	318 ± 8	329 ± 7	-	-
n_1	0.76 ± 0.05	1.15 ± 0.02	0.68 ± 0.01	0.79 ± 0.01
n_2	2.35 ± 0.16	2.15 ± 0.07	-	-

Table S2. Fit results for image correlation spectroscopy functions. Related to Fig. 2.

Parameters for exponential fits (STAR Methods) to the normalized image correlation spectroscopy functions for HP1α and H3K9me3 shown in **Fig. 2C**. Errors represent standard fit errors. The correlation lengths λ_i reflect the radii of the structures in the images and were multiplied by a factor of 2 to obtain the diameters reported in **Fig. 2D**. The parameter values for λ and n depend on each other, which means that the uncertainty for both parameters is larger than reflected by the reported error. The parameters for the second component (a_2, λ_2, n_2) depend on the segmentation settings and should be interpreted with caution.

Fit parameter	GFP-HP1α	MECP2-GFP	GFP-NCL	GFP-NPM	H2B-GFP
τ_D (s)	64 ± 1	233 ± 7	9.8 ± 0.6	18.9 ± 1.4	> 1,000
p	≥ 100	≥ 100	0.64 ± 0.04	≤ 0.01	-

Table S3. Fit results for half-chromocenter and half-nucleolus FRAP. Related to Fig. 5.

Parameters for the confined diffusion fits (STAR Methods) to the FRAP curves shown in **Fig. 5D-H**. Note that τ_D is differently defined than the diffusion time in many other unconfined FRAP models. Permeability values p below 0.01 and above 100 could not be determined as curves showed only subtle changes in these parameter regimes. The permeability for H2B-GFP could not be determined because the protein did not show sufficient diffusive motion during the course of the experiment. Errors represent standard fit errors.

Fit parameter	Cytoplasm	Nucleoplasm	Chromocenter
τ_R (ns)	74 ± 7	117 ± 9	111 ± 8
τ_T (µs) *	0.80 ± 0.09	0.80 ± 0.09	0.80 ± 0.09
τ_{D1} (ms) *	0.33 ± 0.03	0.33 ± 0.03	0.33 ± 0.03
τ_{D2} (ms)	-	35 ± 11	89 ± 18
f_R	0.50 ± 0.03	0.67 ± 0.03	0.80 ± 0.04
f_T *	0.25 ± 0.02	0.25 ± 0.02	0.25 ± 0.02
f_1	1	0.78 ± 0.03	0.67 ± 0.03
α_1	0.70 ± 0.04	0.6	0.6
α_2	-	1.14 ± 0.29	1.23 ± 0.24

Table S4. Fit results for Pol-FCS in living cells. Related to Fig. 6.

Parameters for the rotational-translational diffusion fits (STAR Methods) to the Pol-FCS curves for GFP-HP1α shown in **Fig. 6D** and **6E**. Errors represent standard fit errors.
* f_T, τ_T and τ_{D1} were fitted globally.

Fit parameter	0%	20%	40%	60%	80%
τ_R (ns)	36 ± 5	61 ± 7	107 ± 11	304 ± 20	1199 ± 59
τ_T (µs) *	0.79 ± 0.14	0.79 ± 0.14	0.79 ± 0.14	0.79 ± 0.14	0.79 ± 0.14
τ_D (ms)	0.66 ± 0.09	1.66 ± 0.19	1.25 ± 0.16	2.66 ± 0.37	2.00 ± 0.27
f_R	0.68 ± 0.06	0.53 ± 0.04	0.77 ± 0.04	0.85 ± 0.03	1.14 ± 0.03
f_T *	0.13 ± 0.01	0.13 ± 0.01	0.13 ± 0.01	0.13 ± 0.01	0.13 ± 0.01
α	0.63 ± 0.04	0.64 ± 0.04	0.64 ± 0.04	0.54 ± 0.03	0.54 ± 0.03

Table S5. Fit results for Pol-FCS in glycerol-water mixtures. Related to Fig. 6 and Fig. S6.

Parameters for the rotational-translational diffusion fits (STAR Methods) to the Pol-FCS curves for GFP-HP1 in glycerol-water mixtures shown in **Fig. 6G**. Errors represent standard fit errors.
* f_T and τ_T were fitted globally.
